# Use of 55 PET radiotracers under approval of a Radioactive Drug Research Committee (RDRC)

**DOI:** 10.1186/s41181-020-00110-z

**Published:** 2020-11-11

**Authors:** Isaac M. Jackson, So Jeong Lee, Alexandra R. Sowa, Melissa E. Rodnick, Laura Bruton, Mara Clark, Sean Preshlock, Jill Rothley, Virginia E. Rogers, Leslie E. Botti, Bradford D. Henderson, Brian G. Hockley, Jovany Torres, David M. Raffel, Allen F. Brooks, Kirk A. Frey, Michael R. Kilbourn, Robert A. Koeppe, Xia Shao, Peter J. H. Scott

**Affiliations:** 1 Department of Radiology, University of Michigan, 2276 Medical Science Bldg I, SPC 5610, Ann Arbor, MI 48109 USA; 2 Present Address: Stanford University, Stanford, CA USA; 3 Present Address: Gordon Center for Medical Imaging, Massachusetts General Hospital, Harvard Medical School, Boston, MA USA

**Keywords:** PET imaging, Regulatory oversight, Dosimetry, RDRC, IND, Radiopharmaceuticals, Quality assurance

## Abstract

**Background:**

In the US, EU and elsewhere, basic clinical research studies with positron emission tomography (PET) radiotracers that are generally recognized as safe and effective (GRASE) can often be conducted under institutional approval. For example, in the United States, such research is conducted under the oversight of a Radioactive Drug Research Committee (RDRC) as long as certain requirements are met. Firstly, the research must be for basic science and cannot be *intended* for immediate therapeutic or diagnostic purposes, or to determine the safety and effectiveness of the PET radiotracer. Secondly, the PET radiotracer must be generally recognized as safe and effective. Specifically, the mass dose to be administered must not cause any clinically detectable pharmacological effect in humans, and the radiation dose to be administered must be the smallest dose practical to perform the study and not exceed regulatory dose limits within a 1-year period. In our experience, the main barrier to using a PET radiotracer under RDRC approval is accessing the required information about mass and radioactive dosing.

**Results:**

The University of Michigan (UM) has a long history of using PET radiotracers in clinical research studies. Herein we provide dosing information for 55 radiotracers that will enable other PET Centers to use them under the approval of their own RDRC committees.

**Conclusions:**

The data provided herein will streamline future RDRC approval, and facilitate further basic science investigation of 55 PET radiotracers that target functionally relevant biomarkers in high impact disease states.

**Supplementary Information:**

**Supplementary information** accompanies this paper at 10.1186/s41181-020-00110-z.

## Background

Human use of positron emission tomography (PET) radiotracers in a given country (or member states in the case of the European Union) is required to be conducted under appropriate governmental oversight (Schwarz and Decristoforo 2019; Schwarz et al. 2019). In this paper, we focus upon clinical use of PET radiotracers in the United States, which is regulated by the Food and Drug Administration (FDA) (VanBrocklin 2008; Harapanhalli 2010; Schwarz et al. 2014). However, we expect the regulatory concepts described herein to also hold true in other locations, particularly in light of recent efforts to harmonize PET regulations around the world (Schwarz et al. 2019).

**Fig. 1:**
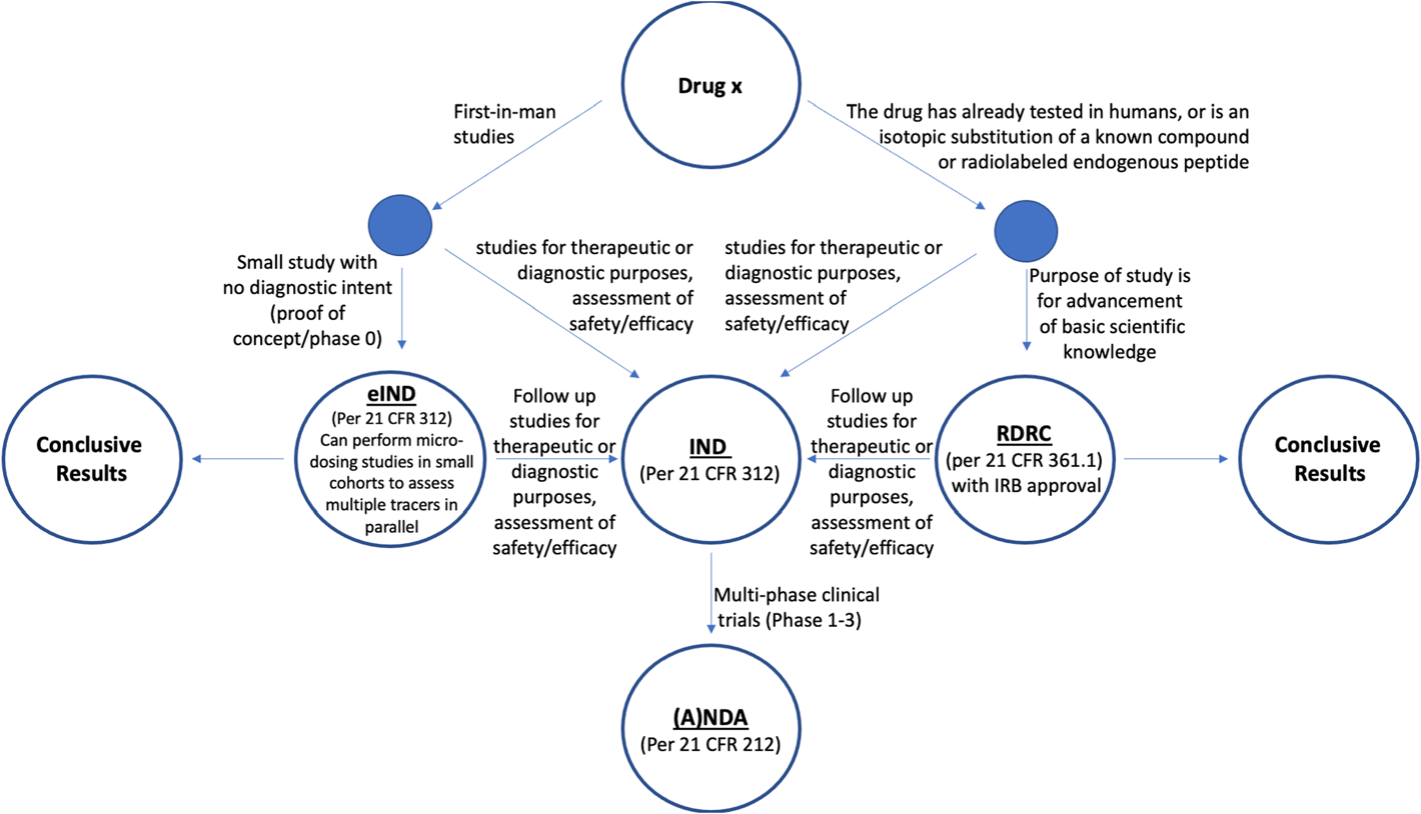
Regulatory Oversight for PET Drugs in the United States

In the US, clinical use of PET radiotracers is conducted under the umbrella of an FDA-approved New Drug Application (NDA) or, in the case of generic PET radiotracers, an Abbreviated New Drug Application (ANDA). Human research is also conducted under governance of the FDA, via three major pathways: i) the Investigational New Drug application (IND), ii) an exploratory IND (eIND), or iii) under the oversight of a Radioactive Drug Research Committee (RDRC) (Suleiman et al. 2006; FDA Guidance for Industry and Researchers: The Radioactive Drug Research Committee: Human Research without an Investigational New Drug Application 2010; Carpenter Jr et al. 2009; Mosessian et al. 2014). The necessary path to approval is dictated by parameters outlined below, as well as the stated purpose of the research in question (Fig. 1).

While the IND and eIND represent the most common pathways to FDA approval for first-in-man studies, some of the requirements (e.g., costly toxicology in two species for an IND) represent significant hurdles to overcome in the application process. One notable solution, as described by Mosessian et al., is to divide labor and preparation for different components of the application between different cores and facilities at a given institution (Mosessian et al. 2014). In contrast, conducting human PET research under RDRC oversight represents a relatively efficient and cost effective path to FDA approval. The concept of the RDRC was introduced in 1975, and committees are charged by the FDA with the responsibility of overseeing PET research at the institutional level. RDRC committees are comprised of at least 5 members and are required to include people with the following expertise:

- Physicians specializing in nuclear medicine;
- Nuclear pharmacists and/or radiochemists that are trained and qualified to formulate radioactive drugs;
- Persons having training in radiation safety and radiation dosimetry;
- Individuals specializing in disciplines pertinent to nuclear medicine (radiology, internal medicine, hematology, endocrinology, radiation therapy, clinical pharmacology, etc.).

In order for a given PET imaging study to be conducted under RDRC approval, the proposed research must meet the following criteria (as described comprehensively in 21 CFR 361.1):

- Stated purpose of the research must fall under the category of basic science, including but not limited to studies of: metabolism, kinetics, biodistribution, pathophysiology, biochemistry, transporter processes, and receptor binding/occupancy.
- The research cannot be *intended* for immediate therapeutic or diagnostic purposes, or to determine the safety and effectiveness of the PET radiotracer, but can have therapeutic/diagnostic *implications*. If at any point research initiated under RDRC approval shifts to directly address these subjects, IND approval must be obtained prior to further studies.
- The protocol must involve less than 30 patients, of age 18 or older (exceptions possible pending special approval), and women of child bearing potential must provide a written statement that they are not pregnant (without exception).
- The PET radiotracer is generally recognized as safe and effective (GRASE). Specifically:
  - ◦ The mass dose to be administered must not cause any clinically detectable pharmacological effect in humans. It is important to note that this generally precludes first-in-human testing of a PET radiotracer from being done under RDRC approval. Notably, RDRC approval can be used for study of radiolabeled endogenous molecules, as well as isotopic substitutions on clinically characterized compounds (i.e; substituting ^18^F for ^19^F on a small molecule ligand that has previously been approved and studied by the FDA, often via IND)
  - ◦ The radiation dose to be administered must be the smallest dose practical to perform a given study. Specifically, the radiation dose to an adult research subject from a single study, or cumulatively from a number of studies, conducted within 1 year may not exceed established regulatory dose limits:
    - Whole Body / Active Blood-Forming Organs / Lens of Eye / Gonads: Single Dose (Effective Dose) = 3 rem (0.03 Sv), Annual & Total Effective Dose Commitment = 5 rem (0.05 Sv);
    - Other Organs: Single Dose = 5 rem (0.05 Sv); Annual and Total Dose Commitment = 15 rem (0.15 Sv).
    The radiation dose to a subject consists of the sum total of all sources of radiation associated with the research protocol, including the PET radiotracer(s), associated x-ray procedures (including CT scans, PET transmission scans etc.) and any follow-up studies.
  - ◦ For research subject under 18 years of age at his or her last birthday, the RDRC regulations require that the radiation limits do not exceed 10% of the radiation dose values given above.
- Other key criteria as described in 21 CFR 361.1 include providing evidence of: Qualified investigators, high quality of drug, research protocol, appropriate licensure to handle radioactive material, and review/approval for work with human subjects (via the Institutional Review Board (IRB)).

If all of these criteria are met, then the research can proceed following RDRC and IRB approval. Research conducted in RDRC studies is considered basic science. Specifically, basic science research is intended to advance scientific knowledge, but not to evaluate safety or efficacy of a PET radiotracer or to make clinical decisions. Failure to meet this, or any of the other RDRC criteria outlined above, necessitates that the research be conducted under an IND or eIND application that has been approved by the U.S. Food and Drug Administration (FDA), as outlined in 21 CFR 312 (Mosessian et al. 2014).

To ensure compliance with the pertinent regulations, FDA vests RDRC committees with oversight responsibility for basic science research conducted at the committee’s institution. The committee reviews and approves research protocols to ensure compliance with RDRC regulations, and submits annual reports to FDA that list committee members and summarize all studies conducted under the committee’s approval in the preceding year. The RDRC committee must also submit a special summary (Form FDA 2915) for any approved study involving > 30 research subjects (Suleiman et al. 2006).

Conducting human PET imaging under RDRC approval represents a straightforward and economical pathway to clinical use, particularly since there is no requirement for resource intensive pharmacology-toxicology studies. In our experience, the main barrier to using a PET radiotracer for basic science under RDRC approval is actually accessing the required information about pharmacological dose/mass and radioactive dose. For a given radiotracer this information can either come from the peer-reviewed scientific literature or other valid data, often in the form of a signed letter from an institution already working with the radiotracer in question. In an effort to remove barriers to those wishing to conduct clinical PET research under RDRC, herein we provide pharmacological dose and radioactive dosimetry details for 55 such PET radiotracers that will enable other PET Centers to use them under the approval of their own RDRC committees, eliminating the need to obtain a specific signed letter on a case-by-case basis. The article is made available Open Access in an attempt to further improve accessibility for our imaging colleagues, and we encourage other PET Centers with large clinical radiotracer portfolios to publish sister articles in the near future.

## Methods

### Radiosyntheses

PET radiotracers were commercially available, or synthesized according to the literature radiosyntheses referenced in Table 1 or novel radiosyntheses described in the Supporting Information. Production and quality control of all radiotracers was conducted according to current Good Manufacturing Practice (cGMP) using the guidelines outlined in the US Pharmacopeia, (USP < 823> Positron Emission Tomography Drugs for Compounding, Investigational, and Research Uses 2020).

**Table 1:** Radiotracers used clinically at the University of Michigan

Radiotracer	Abbreviation	Application	Radiosynthesis	Dosimetry	Historical Imaging
**[**^**11**^**C]Radiotracers**					
[^11^C]Acetate	[^11^C]ACE	Metabolism	Runkle et al. 2011	Seltzer et al. 2004	Duvernoy et al. 2016
[^11^C]Aminocyclohexane carboxylic acid	[^11^C]ACHC	Amino acid transport	Koeppe et al. 1990a	Washburn et al. 1982^a^	Koeppe et al. 1990a
1-[^11^C]Methyl-4- piperidinyl n-butyrate	[^11^C]BMP	Butyrlcholinesterase	Snyder et al. 2001	Virta et al. 2008	Kuhl et al. 2006
[^11^C]Butanol	[^11^C]BUT	Blood flow	See Supporting Information	See Supporting Information	^b^; Quarles et al. 1993
[^11^C]Carfentanil	[^11^C]CFN	Mu opioid receptors	Blecha et al. 2017	Newberg et al. 2009	Zubieta et al. 2000
[^11^C]Choline	[^11^C]CHO	Choline biochemistry	Shao et al. 2014	Tolvanen et al. 2010	Piert et al. 2009
[^11^C]DASB	[^11^C]DASB	Serotonin transporter	Shao et al. 2014	Lu et al. 2004	Albin et al. 2008
[^11^C]Dihydrotetrabenazine	[^11^C]DTBZ	Vesicular monoamine transporter 2 (VMAT2)	Shao et al. 2014	Murthy et al. 2008	Koeppe et al. 1995
[^11^C]Epinephrine	[^11^C]EPI	Norepinephrine Transporter (NET)	Chakraborty et al. 1993	Wrobel et al. 1997	Münch et al. 2000
[^11^C]Flumazenil	[^11^C]FMZ	GABA_A_ Receptors	Shao et al. 2011a	Laymon et al. 2012	Koeppe et al. 1991
[^11^C]*meta*-Hydroxyephedrine	[^11^C]HED	NET, Sympathetic nervous system	Shao et al. 2014	Wrobel et al. 1997 and Supporting Information	Duvernoy et al. 2016
[^11^C]LY2795050	[^11^C]LY2795050	Kappa opioid receptors	Yang et al. 2018	See Supporting Information	^b^; Naganawa et al. 2015
[^11^C]Methionine	[^11^C]MET	Amino acid	Shao et al. 2014	Deloar et al. 1998	Miller et al. 2019
[^11^C]Methoxytetrabenazine	[^11^C]MTBZ	VMAT2	DaSilva et al. 1993a	Wrobel et al. 1997	Vander Borght et al. 1995
[^11^C]Methylphenidate	[^11^C]MPH	Dopamine transporter	Moran et al. 2010	See Supporting Information	Albin et al. 2009
[^11^C]N-Methylpiperidinyl benzilate	[^11^C]NMBP	mAChR	Mulholland et al. 1995	Mulholland et al. 1995	Zubieta et al. 2001
[^11^C]OMAR/[^11^C]JHU 75528	[^11^C]OMAR	Cannabinoid 2 receptors	Shao et al. 2015	Wong et al. 2010	Wong et al. 2010
[^11^C]Palmitate	[^11^C]PALM	Fatty acid metabolism	Runkle et al. 2011	Christensen et al. 2017	^b^; de Jong et al. 2009
[^11^C]PBR28	[^11^C]PBR28	Translocator protein 18 kDa (TSPO)	Shao et al. 2014	Brown et al. 2007	^b^ Kreisl et al. 2016
[^11^C]Pittsburgh Compound B	[^11^C]PiB	Amyloid plaques	Shao et al. 2014	O’Keefe et al. 2009	Burke et al. 2011
[^11^C]((*E*)-N-(3-iodoprop-2-enyl)-2β-(4′-tolyl) nortropane)	[^11^C]PE2I	Dopamine transporter	Dollé et al. 2000; Halldin et al. 2003	Ribeiro et al. 2007	^b;^ Halldin et al. 2003
[^11^C]Phenylephrine	[^11^C]PHEN	NET	Del Rosario et al. 1996	Wrobel et al. 1997	Raffel et al. 1996
(*R*)-[N-Methyl-^11^ C]PK11195	[^11^C]PK11195	TSPO	Alves et al., 2013	Hirvonen et al. 2010	Junck et al. 1989
[^11^C]PMP	[^11^C]PMP	Acetylcholinesterase	Shao et al. 2014	See Supporting Information	Kuhl et al. 1999
[^11^C]Raclopride	[^11^C]RAC	Dopamine D_2_ receptors	Shao et al. 2014	Ribeiro et al. 2005	Scott et al. 2006
[^11^C]Ro-54,864	[^11^C]Ro-54,864	TSPO	Watkins et al. 1988	see Supporting Information^a^	Junck et al. 1989
[^11^C]Sarcosine	[^11^C]SARC	Sarcosine biochemistry	Piert et al. 2017	Piert et al. 2017	Piert et al. 2017
[^11^C]Scopolamine	[^11^C]SCOP	mAChR	Mulholland et al. 1988	Frey et al. 1992	Frey et al. 1992
[^11^C]Tetrabenazine	[^11^C]TBZ	VMAT2	DaSilva et al. 1993b	DaSilva et al. 1994	Kilbourn et al. 1993
[^11^C]Tropanylbenzilate	[^11^C]TRB	mAChR	Mulholland et al. 1992	Mulholland et al. 1992	Koeppe et al. 1994
[^11^C]WAY-100365	[^11^C]WAY	5-HT_1A_ Receptor	Krasikova et al. 2009	Parsey et al. 2005	Mickey et al. 2008
**[**^**18**^**F]Radiotracers**					
[^18^F]Flortaucipir	[^18^F]AV1451; [^18^F]T807; Tauvid	Tau	^c^; Mossine et al. 2017	Choi et al. 2016	Drake et al. 2019; Kramer et al. 2020
3-(1,4-Diazabicyclo[3.2.2] nonan-4-yl)-6-[^18^F]fluoro-dibenzo[b,d]thiophene 5,5-dioxide	[^18^F]JHU82132, [^18^F]ASEM	α7 nicotinic acetylcholine receptor (nAChR)	Gao et al. 2013 and Supporting Information	Wong et al. 2014 and Supporting Information	^b^; Wong et al. 2014
Fluciclovine (anti-1-Amino-3-^18^F-fluorocyclobutane-1-carboxylic acid)	[^18^F]FACBC; Auxumin	Amino acid transport	^c^; Sörensen et al. 2013	Nye et al. 2007; McParland et al. 2010	^b^; Songmen et al. 2019
[^18^F]Fluoroazomycin arabinoside	[^18^F]FAZA	Tumor hypoxia	Shao et al. 2011b	Savi et al. 2017	Beck et al., 2007
2-[^18^F]Fluoro-2-deoxy-D-glucose	[^18^F]FDG	Glucose metabolism	Richards and Scott 2012; Sowa et al. 2018	Srinivasan et al. 2020	Koeppe et al. 2005
[^18^F]6-Fluoro-L-DOPA	[^18^F]FDOPA	Dopamine	See Supporting Information, Mossine et al. 2019, 2020	Kaushik et al. 2013; Mejia et al. 1991	Minn et al. 2009
[^18^F]-Fluoroethoxy- benzovesamicol	[^18^F]FEOBV	Vesicular acetylcholine transporter	Shao et al. 2011b	Petrou et al. 2014	Petrou et al. 2014
[^18^F]Fluorocholine	[^18^F]FCH	Choline biochemistry	Rodnick et al. 2013	DeGrado et al. 2002; Fabbri et al. 2014	Davenport et al. 2020
[^18^F]Florbetapir	Amyvid; [^18^F]AV45	Amyloid plaques	c	Joshi et al. 2014	Frey and Koeppe 2016
[^18^F]-Fluoro-3′-deoxy-3′-L-fluorothymidine	[^18^F]FLT	Cellular proliferation	Shao et al. 2011b	Vesselle et al. 2003; Mendes et al. 2018	Bertagna et al. 2013
[^18^F]Flubatine	[^18^F]FLBT	α_4_β_2_ nAChR	Hockley et al. 2013	Kranz et al. 2016	Sattler et al. 2012
[^18^F]Flutemetamol	Vizamyl, [^18^F]GE67	Amyloid plaques	^c^; Snellman et al. 2014	Koole et al. 2009	Frey and Koeppe 2016
[^18^F]Fluoromisonidazole	[^18^F]FMISO	Tumor hypoxia	^d^; Riss et al. 2012	Graham et al. 1997	Bruehlmeier et al. 2004
[^18^F]Fluoropropyl-dihydrotetrabenazine	[^18^F]FP-TBZ, [^18^F]AV133	VMAT2	Lin et al. 2010	Lin et al. 2010	Kilbourn and Koeppe 2019
[^18^F]GBR13119 /[^18^F]GBR12909	[^18^F]GBR	DAT	Haka and Kilbourn 1988, 1990	Kilbourn et al. 1989	Koeppe et al. 1990b
4-[^18^F]Fluoro-*m*-hydroxyphenethylguanidine	[^18^F]MHPG	NET	Raffel et al. 2018	Raffel et al. 2018	Raffel et al. 2018
2′-Methoxyphenyl-(N-2′-pyridinyl)-p-^18^F-fluoro-benzamidoethylpiperazine	[^18^F]MPPF	5-HT_1A_ Receptor	Shao et al. 2011b	See Supporting Information	^b^; Aznavour and Zimmer 2007
[^18^F]Sodium Fluoride	[^18^F]NaF	Bone imaging	Shao et al. 2011b	Segall et al. 2010; Silveira et al. 2010	Wong and Piert 2013
[^18^F]N-Methyl Lansoprazole	[^18^F]NML	Tau	Kramer et al. 2020	Kramer et al. 2020	Kramer et al. 2020
4-[^18^F]Fluoro-*p*-hydroxyphenethylguanidine	[^18^F]PHPG	NET	Raffel et al. 2018	Raffel et al. 2018	Raffel et al. 2018
**Other Radiotracers**					
[^13^N]Ammonia	[^13^N]NH_3_	Blood flow	Scott 2012	Yi et al. 2015	Beanlands et al. 1994
[^15^O]Water	[^15^O]H_2_O	Blood flow	Dick and Watkins 2015	Brihaye et al. 1995	Minoshima et al. 1993
[^68^Ga]DOTATATE	NETSPOT	Somatostatin receptors	NETSPOT prescribing information 2016	Walker et al. 2013	^b^; Fallahi et al. 2019
[^68^Ga]PSMA-HBEDCC	[^68^Ga]PSMA-11	Prostate specific membrane antigen	See Supporting Information; Rodnick et al. 2020	Afshar-Oromieh et al. 2016; Sandgren et al. 2019	Rodnick et al. 2020

^a^Historical dosimetry data is no longer extant. Biodistribution data are provided to enable estimation of dosimetry; ^b^ UM Imaging data not yet published; ^c^ Commercially available under an approved (A)NDA; ^d^ Commercially available under an IND

### Dosimetry

Radiation-absorbed-dose estimates can either be obtained from literature sources or determined using the OLINDA/EXM 1.0 software package (Stabin et al. 2005). Table 1 provides literature sources of dosimetry wherever available. For any radiotracers where literature dosimetry is unavailable, dosimetry is provided in the Supporting Information.

### Imaging

Research PET scans have been conducted since the first PET scanner was installed at the University of Michigan (UM) in the 1980s. Historical examples of imaging studies mostly conducted at our Center with the various radiotracers are provided in Table 1, including practical information on both scanning protocols and image kinetic analysis. Injected dose (MBq), mass dose limits (μg) and historical numbers of subjects scanned are provided in Table 2.

**Table 2:** Dosing information

Radiotracer	Injected Dose (MBq)	Mass dose limit	Number of subjects scanned	Clinically detectable pharmacological effects in humans
**[**^**11**^**C]Radiotracers**				
[^11^C]ACE	740	None	475	No
[^11^C]ACHC	740	≤5000 μg/subject	2	No
[^11^C]BMP	444	≤4625 μg/subject	65	No
[^11^C]BUT	555	≤125 μg/kg	0^a^	a
[^11^C]CFN	555	≤0.03 μg/kg	1492	No
[^11^C]CHO	592	None	44	No
[^11^C]DASB	666	≤8 μg/subject	179	No
[^11^C]DTBZ	555	≤50 μg/subject	1823	No
[^11^C]EPI	740	< 9 μg/subject epinephrine & ≤1 μg/subject norepinephrine precursor	96	No
[^11^C]FMZ	370	≤50 μg/subject	668	No
[^11^C]HED	666	≤50 μg/subject^b^	643	No
[^11^C]LY2795050	555	≤10 μg/subject	0^c^	c
[^11^C]MET	444	None	129	No
[^11^C]MTBZ	580	≤10 μg/subject	6	No
[^11^C]MPH	666	≤25 μg/subject	170	No
[^11^C]NMBP	740–1480	≤127 μg/subject^d^	59	No
[^11^C]OMAR	666	0.14 μg/kg	0^e^	e
[^11^C]PALM	740	None	8	No
[^11^C]PBR28	666	≤10 μg/subject	34	No
[^11^C]PiB	666	≤13 μg/subject	592	No
[^11^C]PE2I	555	≤6.3 μg/subject	1	No
[^11^C]PHEN	740	≤6800 μg/subject	29	No
[^11^C]PK11195	888	≤420 μg/subject	118	No
[^11^C]PMP	555	≤200 μg/subject	801	No
[^11^C]RAC	555	≤50 μg/subject	627	No
[^11^C]Ro-54,864	555	≤160 μg/subject	6	No
[^11^C]SARC	592	None	20	No
[^11^C]SCOP	1480	≤50 μg/subject	14	No
[^11^C]TBZ	1018	≤10 μg/subject	2	No
[^11^C]TRB	1110	≤31 μg/subject	26	No
[^11^C]WAY-100365	555	≤15 μg/subject	51	No
**[**^**18**^**F]Radiotracers**				
[^18^F]AV1451	370	≤20 μg/subject	92	No
[^18^F]ASEM	370	≤0.67 μg/subject	1	No
Auxumin	370	≤20 μg/subject	228^f^	No
[^18^F]FAZA	296	≤3.5 μg/subject	14	No
[^18^F]FDG	185–296	None	6804	No
[^18^F]FDOPA	148	≤15 μg/subject	0^g^	g
[^18^F]FEOBV	296	≤1.23 μg/subject	308	No
[^18^F]FCH	222	≤100 μg/subject	67	No
Amyvid	370	≤50 μg/subject	222	No
[^18^F]FLT	370	≤20 μg/subject	8	No
[^18^F]FLBT	296	≤0.02 μg/kg	92	No
Vizamyl	370	≤20 μg/subject	11	No
[^18^F]FMISO	370	≤15 μg/subject	8	No
[^18^F]FP-TBZ	370	≤7.5 μg/subject	23	No
[^18^F]GBR	148	≤900 μg/subject	2	No
[^18^F]MHPG	241	≤10 μg/subject	17	No
[^18^F]MPPF	259	≤2 μg/subject	34	No
[^18^F]NaF	222	None	9	No
[^18^F]NML	370	≤10 μg/subject	6	No
[^18^F]PHPG	241	≤10 μg/subject	15	No
**Other Radiotracers**				
[^13^N]NH_3_	740	None	1472	No
[^15^O]Water	555	None	1153	No
NETSPOT	200	≤40 μg/subject	981^f^	No
[^68^Ga]PSMA-11	185	≤10 μg/subject	751^f^	No

^a^[^11^C]Butanol is validated for clinical production but studies have not yet commenced. We do not expect clinically detectable pharmacological effects as the mass limit (≤125 μg/kg) was selected since it is 1000 times below the NOAEL (125 mg/kg, see: Wagner 2005); ^b^ combined mass of HED and metaraminol precursor must be ≤50 μg/subject; ^c^ [^11^C]LY2795050 is validated for clinical production but studies have not yet commenced at UM. We do not expect clinically detectable pharmacological effects as the mass limit (≤10 μg/subject) has been used without significant adverse events at other institutions (see: Naganawa et al. 2015); ^d^ See published limits (Yoshida et al. 1998); ^e^ [^11^C]OMAR is validated for clinical production but studies have not yet commenced at UM. We do not expect clinically detectable pharmacological effects as the mass limit (≤0.14 μg/kg) has been used without significant adverse events at other institutions (see: Wong et al. 2010); ^f^ Includes subjects numbers scanned for clinical care and research; ^g^ [^18^F]FDOPA is validated for clinical production but studies have not yet commenced. We do not expect clinically detectable pharmacological effects as the mass limit (≤15 μg/subject) is significantly less than administered masses historically used when employing the electrophilic synthesis of [^18^F]FDOPA (13 mg/62 kg subject, see: Chevalme et al. 2007)

## Discussion

At the University of Michigan we have a long history of using PET radiotracers in clinical research studies (using both the RDRC and IND mechanisms). Detailed information for 55 such radiotracers is provided in Table 1, including references for radiosyntheses and dosimetry available in the peer-reviewed literature. Synthesis ([^11^C]butanol, [^18^F]ASEM, [^18^F]FDOPA, [^68^Ga]PSMA-11) and dosimetry ([^18^F]ASEM, [^11^C]butanol, [^11^C]HED, [^11^C]LY2795050, [^11^C]MPH, [^18^F]MPPF, [^11^C]PMP, [^11^C]RO-54864) information that has not previously been published is provided in the Supporting Information associated with this article. Pharmacological dose and radioactivity dosing information for the PET drugs is also provided (Table 2), along with historical numbers of administrations to subjects at the University of Michigan PET Center. Rationale for those radiotracers without mass dose limits is provided in the Supporting Information.

As noted above, a study conducted under RDRC oversight cannot exceed 30 subjects without special provisions. The PET drugs corresponding to some of the larger numbers of subjects discussed herein have been used in numerous different RDRC studies over the course of many years (and decades in some instances). In the event any given study exceeded 30 research subjects, the RDRC committee filed a special summary (Form FDA 2915). At the doses specified, no pharmacological or physiological changes were observed after intravenous administration of any of the PET drugs, and the basic science studies were conducted without exceeding any regulatory radiation dose limits. All scans have been reported to the US FDA in the annual RDRC reports required by the agency.

## Conclusion

While an IND (or eIND) is the dominant route to FDA approval for first-in-man studies, collection of the requisite data and preparation of the application can be a daunting and resource intensive task. Proceeding under approval of a Radioactive Drug Research Committee therefore represents an attractive mechanism for clinical studies of compounds that have (a) already been studied in man and (b) are well characterized in terms of pharmacology and dosimetry. Initiation of a new study for such an established compound is contingent upon access to mass dose and dosimetry data. The data provided herein will streamline future RDRC approval, and facilitate further basic science investigation of 55 PET drugs that target functionally relevant biomarkers in high impact disease states.

## Supplementary Information

**Additional file 1.**

## Data Availability

The datasets used in the current paper are available from the corresponding author on reasonable request.

## Abbreviations

ANDA: Abbreviated new drug application
Bq: Becquerels
cGMP: Current good manufacturing practice
eIND: Exploratory IND
FDA: Food and Drug Administration
IND: Investigational new drug
NDA: New drug application
PET: Positron emission tomography
QC: Quality control
RDRC: Radioactive drug research committee
USP: United States Pharmacopeia

## References

[CR1] Afshar-Oromieh A, Hetzheim H, Kübler W, Kratochwil C, Giesel FL, Hope TA, Eder M, Eisenhut M, Kopka K, Haberkorn U (2016). Radiation dosimetry of ^68^Ga-PSMA-11 (HBED-CC) and preliminary evaluation of optimal imaging timing. Eur J Nucl Med Mol Imaging.

[CR2] Albin R, Koeppe R, Bohnen N, Wernette K, Kilbourn M, Frey K (2008). Spared caudal brainstem SERT binding in early Parkinson disease. J Cereb Blood Flow Metab.

[CR3] Albin RL, Koeppe RA, Wernette K, Zhuang W, Nichols T, Kilbourn MR, Frey KA (2009). Striatal [^11^C]dihydrotetrabenazine and [^11^C]methylphenidate binding in Tourette syndrome. Neurology.

[CR4] Alves VH, Abrunhosa AJ, Castelo-Branco M. Optimisation of synthesis, purification and reformulation of (R)-[N-Methyl-11C]PK11195 for in vivo PET imaging studies. In Proceedings of the 2013 IEEE 3rd Portuguese Meeting in Bioengineering (ENBENG), Braga, Portugal, 20–23 February 2013; pp. 1–5.

[CR5] Aznavour N, Zimmer L (2007). [^18^F]MPPF as a tool for the in vivo imaging of 5-HT1A receptors in animal and human brain. Neuropharmacology.

[CR6] Beanlands RS, Muzik O, Hutchins GD, Wolfe ER, Schwaiger M (1994). Heterogeneity of regional nitrogen 13-labeled ammonia tracer distribution in the normal human heart: comparison with rubidium 82 and copper 62-labeled PTSM. J Nucl Cardiol.

[CR7] Beck R, Roper B, Carlsen JM, Huisman MC, Lebschi JA, Andratschke N, Picchio M, Souvatzoglou M, Machulla H-J, Piert M (2007). Pretreatment 18F-FAZA PET Predicts Success of Hypoxia-Directed Radiochemotherapy Using Tirapazamine. J Nucl Med..

[CR8] Bertagna F, Biasiotto G, Giubbini R (2013). The role of F-18-fluorothymidine PET in oncology. Clin Transl Imaging.

[CR9] Blecha JE, Henderson BD, Hockley BG, VanBrocklin HF, Zubieta J-K, DaSilva AF, Kilbourn MR, Koeppe RA, Scott PJH, Shao X (2017). An updated synthesis of [^11^C]Carfentanil for positron emission tomography (PET) imaging of the μ-opioid receptor J. Labelled Comp Radiopharm.

[CR10] Brihaye C, Depresseux JC, Comar D (1995). Radiation dosimetry for bolus administration of oxygen-15-water. J Nucl Med.

[CR11] Brown AK, Fujita M, Fujimura Y, Liow J-S, Stabin M, Ryu YH, Imaizumi M, Hong J, Pike VW, Innis RB (2007). Radiation dosimetry and biodistribution in monkey and man of ^11^C-PBR28: a PET radioligand to image inflammation. J Nucl Med.

[CR12] Bruehlmeier M, Roelcke U, Schubiger PA, Ametamey SM (2004). Assessment of hypoxia and perfusion in human brain tumors using PET with ^18^F-fluoromisonidazole and ^15^O-H_2_O. J Nucl Med.

[CR13] Burke JF, Albin RL, Koeppe RA, Giordani B, Kilbourn MR, Gilman S, Frey KA (2011). Assessment of mild dementia with amyloid and dopamine terminal positron emission tomography. Brain.

[CR14] Carpenter AP, Pontecorvo MJ, Hefti FF, Skovronsky DM (2009). The use of the exploratory IND in the evaluation and development of ^18^F-PET radiopharmaceuticals for amyloid imaging in the brain: a review of one company's experience. Q J Nucl Med Mol Imaging.

[CR15] Chakraborty PK, Gildersleeve DL, Jewett DM, Toorongian SA, Kilbourn MR, Schwaiger M, Wieland DM (1993). High yield synthesis of high specific activity R-( −)-[^11^C]epinephrine for routine PET studies in humans. Nucl Med Biol.

[CR16] Chevalme Y-M, Montravers F, Vuillez J-P, Zanca M, Fallais C, Oustrin J, Talbot J-N (2007). FDOPA-(18F): a PET radiopharmaceutical recently registered for diagnostic use in countries of the European Union. Braz Arch Biol Technol.

[CR17] Choi JY, Lyoo CH, Lee JH, Cho H, Kim KM, Kim JS, Ryu YH (2016). Human radiation dosimetry of [^18^F]AV-1451(T807) to detect tau pathology. Mol Imaging Biol.

[CR18] Christensen NL, Jakobsen S, Schacht AC, Munk OL, Alstrup AKO, Tolbod LP, Harms HJ, Nielsen S, Gormsen LC (2017). Whole-body biodistribution, dosimetry, and metabolite correction of [^11^C]Palmitate: a PET tracer for imaging of fatty acid metabolism. Mol Imaging.

[CR19] DaSilva JN, Carey JE, Sherman PS, Pisani TJ, Kilbourn MR (1994). Characterization of [^11^C]tetrabenazine as an in vivo radioligand for the vesicular monoamine transporter. Nucl Med Biol.

[CR20] DaSilva JN, Kilbourn MR, Mangner TJ (1993). Synthesis of a [^11^C]methoxy derivative of α-dihydrotetrabenazine: a radioligand for studying the vesicular monoamine transporter. Appl Radiat Isot.

[CR21] DaSilva JN, Kilbourn MR, Mangner TJ (1993). Synthesis of a [^11^C]tetrabenazine, a vesicular monoamine uptake inhibitor, for PET imaging studies. Appl Radiat Isot.

[CR22] Davenport MS, Montgomery JS, Kunju LP, Siddiqui J, Shankar PR, Rajendiran T, Shao X, Lee E, Denton B, Barnett C, Piert M (2020). ^18^F-choline PET/mpMRI for detection of clinically significant prostate cancer: part 1. Improved risk stratification for MRI-guided transrectal prostate biopsies. J Nucl Med.

[CR23] de Jong HW, Rijzewijk LJ, Lubberink M, van der Meer RW, Lamb HJ, Smit JW, Diamant M, Lammertsma AA (2009). Kinetic models for analysing myocardial [^11^C]palmitate data. Eur J Nucl Med Mol Imaging.

[CR24] DeGrado TR, Reiman RE, Price DT, Wang S, Coleman RE (2002). Pharmacokinetics and radiation dosimetry of ^18^F-fluorocholine. J Nucl Med.

[CR25] Del Rosario RB, Jung Y-W, Caraher J, Chakraborty PK, Wieland DM (1996). Synthesis and preliminary evaluation of [^11^C]-(−)-phenylephrine as a functional heart neuronal PET agent. Nucl Med Biol.

[CR26] Deloar HM, Fujiwara T, Nakamura T, Itoh M, Imai D, Miyaka M, Watanuki S (1998). Estimation of internal absorbed dose of L-[methyl-^11^C]methionine using whole-body positron emission tomography. Eur J Nucl Med Mol Imaging.

[CR27] Dick DW, Watkins GL, Scott PJH (2015). Synthesis of oxygen-15 water ([^15^O]H_2_O). Radiochemical syntheses volume 2: further radiopharmaceuticals for positron emission tomography and new strategies for their production.

[CR28] Dollé F, Bottlaender M, Demphel S, Emond P, Fuseau C, Coulon C, Ottaviani M, Valette H, Loch C, Halldin C, Mauclare L, Guilloteau D, Maziere B, Crouzel C (2000). Highly efficient synthesis of [^11^C]PE2I, a selective radioligand for the quantification of the dopamine transporter using PET. J Labelled Comp Radiopharm.

[CR29] Drake LR, Pham JM, Desmond TJ, Mossine AV, Lee SJ, Kilbourn MR, Koeppe RA, Brooks AF, Scott PJH (2019). Identification of AV-1451 as a weak, nonselective inhibitor of monoamine oxidase. ACS Chem Neurosci.

[CR30] Duvernoy CS, Raffel DM, Swanson SD, Jaiswal M, Mueller G, Ibrahim E-S, Pennathur S, Plunkett C, Stojanovska J, Brown MB, Pop-Busui R (2016). Left ventricular metabolism, function, and sympathetic innervation in men and women with type 1 diabetes. J Nucl Cardiol.

[CR31] Fabbri C, Galassi R, Moretti A, Sintuzzi E, Mautone V, Sarti G, Strigari L, Benassi M, Matteucci F (2014). Radiation dosimetry of ^18^F-flurocholine PET/CT studies in prostate cancer patients. Phys Med.

[CR32] Fallahi B, Manafi-Farid R, Eftekhari M, Fard-Esfahani A (2019). Diagnostic efficiency of ^68^Ga-DOTATATE PET/CT as compared to ^99m^Tc-Octreotide SPECT/CT and conventional morphologic modalities in neuroendocrine tumors. Asia Ocean J Nucl Med Biol.

[CR33] FDA Guidance for Industry and Researchers: The Radioactive Drug Research Committee: Human Research without an Investigational New Drug Application. 2010. https://www.fda.gov/media/76286/download. Accessed 7 Jul 2020.

[CR34] Frey KA, Koeppe RA (2016). PET amyloid analyses. J Nucl Med.

[CR35] Frey KA, Koeppe RA, Mulholland GK, Jewett D, Hichwa R, Ehrenkaufer RLE, Carey JE, Wieland DM, Kuhl DE, Agranoff BW (1992). In vivo muscarinic Cholingeric receptor imaging in human brain with [^11^C]scopolamine and positron emission tomography. J Cereb Blood Flow Metab.

[CR36] Gao Y, Kellar KJ, Yasuda RP, Tran T, Xiao Y, Dannals RF, Horti AG (2013). Derivatives of dibenzothiophene for PET imaging of a7-nicotinic acetylcholine receptors. J Med Chem.

[CR37] Graham MM, Peterson LM, Link JM, Evans ML, Rasey JS, Koh W-J, Caldwell JH, Krohn KA (1997). Fluorine-18-Fluoromisonidazole radiation dosimetry in imaging studies. J Nucl Med.

[CR38] Haka MS, Kilbourn MR (1988). Synthesis of [^18^F]GBR 13119, a presynaptic dopamine uptake antagonist. Appl Radiat Isot.

[CR39] Haka MS, Kilbourn MR (1990). Synthesis of [^18^F]GBR 12909, a dopamine reuptake inhibitor. J Labelled Comp Radiopharm.

[CR40] Halldin C, Erixon-Lindroth N, Pauli S, Chou Y-H, Okubo Y, Karlsson P, Lundkvist C, Olsson H, Guilloteau D, Emond P, Farde L (2003). [^11^C]PE2I: a highly selective radioligand for PET examination of the dopamine transporter in monkey and human brain. Eur J Nucl Med Mol Imaging.

[CR41] Harapanhalli RS (2010). Food and Drug Administration requirements for testing and approval of new radiopharmaceuticals. Semin Nucl Med.

[CR42] Hirvonen J, Roivainen A, Virta J, Helin S, Någren K, Rinne JO (2010). Human biodistribution and radiation dosimetry of ^11^C-(*R*)-PK11195, the prototypic PET ligand to image inflammation. Eur J Nucl Med Mol Imaging.

[CR43] Hockley BG, Stewart MN, Sherman P, Quesada C, Kilbourn MR, Albin RL, Scott PJH (2013). (−)-[^18^F]Flubatine: evaluation in rhesus monkeys and a report of the first fully automated radiosynthesis validatedfor clinical use. J Labelled Comp Radiopharm.

[CR44] Joshi AD, Pontecorvo MJ, Adler L, Stabin MG, Skovronsky DM, Carpenter AP, Mintun MA (2014). Radiation dosimetry of florbetapir F 18. EJNMMI Res..

[CR45] Junck L, Olson JM, Ciliax BJ, Koeppe RA, Watkins GL, Jewett DM, McKeever PE, Wieland DM, Kilbourn MR, Starosta-Rubinstein S, Mancini WR, Kuhl DE, Greenberg HS, Young AB (1989). PET imaging of human gliomas with ligands for the peripheral benzodiazepine binding site. Ann Neurol.

[CR46] Kaushik A, Jaimini A, Tripathi M, D'Souza M, Sharma R, Mishra AK, Mondal A, Dwarakanath BS (2013). Estimation of patient dose in ^18^F-FDG and ^18^F-FDOPA PET/CT examinations. J Can Res Ther.

[CR47] Kilbourn MR, Carey JE, Koeppe RA, Haka MS, Hutchins GD, Sherman PS, Kuhl DE (1989). Biodistribution, dosimetry, metabolism and monkey PET studies of [^18^F]GBR 13119. Imaging the dopamine uptake system in vivo. Nucl Med Biol.

[CR48] Kilbourn MR, DaSilva JN, Frey KA, Koeppe RA, Kuhl DE (1993). In vivo imaging of vesicular monoamine transporters in human brain using [^11^C]Tetrabenazine and positron emission tomography. J Neurochem.

[CR49] Kilbourn MR, Koeppe RA (2019). Classics in neuroimaging: radioligands for the vesicular monoamine transporter 2. ACS Chem Neurosci.

[CR50] Koeppe RA, Frey KA, Mulholland GK, Kilbourn MR, Buck A, Lee KS, Kuhl DE (1994). [^11^C]Tropanyl Benzilate-binding to muscarinic cholinergic receptors: methodology and kinetic modeling alternatives. J Cereb Blood Flow Metab.

[CR51] Koeppe RA, Frey KA, Vander Borght TM, Kilbourn MR, Jewett DM, Lee LC, Kuhl DE (1995). Kinetic evaluation of α-[C-11]dihydrotetrabenazine (DTBZ): a PET ligand for assessing the vesicular monoamine transporter. J Nucl Med.

[CR52] Koeppe RA, Gilman S, Joshi A, Liu S, Little R, Junck L, Heumann M, Frey KA, Albin RL (2005). ^11^C-DTBZ and ^18^F-FDG PET measures in differentiating dementias. J Nucl Med.

[CR53] Koeppe RA, Holthoff VA, Frey KA, Kilbourn MR, Kuhl DE (1991). Compartmental analysis of [^11^C]flumazenil kinetics for the estimation of ligand transport rate and receptor distribution using positron emission tomography. J Cereb Blood Flow Metab.

[CR54] Koeppe RA, Kilbourn MR, Frey KA, Penney JB, Haka MS, Kuhl DE (1990). Imaging and kinetic modeling of [F-18]GBR 12909, a dopamine uptake inhibitor. J Nucl Med.

[CR55] Koeppe RA, Mangner T, Betz AL, Shulkin BL, Allen R, Kollros P, Kuhl DE, Agranoff BW (1990). Use of [^11^C]Aminocyclohexanecarboxylate for the measurement of amino acid uptake and distribution volume in human brain. J Cereb Blood Flow Metab.

[CR56] Koole M, Lewis DM, Buckley C, Nelissen N, Vandenbulcke M, Brooks DJ, Vandenberghe R, Van Laere K (2009). Whole-body biodistribution and radiation dosimetry of 18F-GE067: a radioligand for in vivo brain amyloid imaging. J Nucl Med.

[CR57] Kramer V, Brooks AF, Haeger A, Kuljs RO, Rafique W, Koeppe RA, Raffel DM, Frey KA, Amaral H, Scott PJH, Riss PJ (2020). Evaluation of [^18^F]N-methyl-lansoprazole as a tau PET imaging agent in first-in-human studies. ACS Chem Neurosci.

[CR58] Kranz M, Sattler B, Tiepot S, Wilke S, Deuther-Conrad W, Donat CK, Fischer S, Patt M, Schildan A, Patt J, Smits R, Hoepping A, Steinbach J, Sabri O, Brust P (2016). Radiation dosimetry of the α_4_β_2_ nicotinic receptor ligand (+)-[^18^F]flubatine, comparing preclinical PET/MRI and PET/CT to first-in-human PET/CT results. EJNMMI Physics.

[CR59] Krasikova RN, Andersson J, Truong P, Nag S, Shchukina EV, Halldin C (2009). A fully automated one-pot synthesis of [carbonyl-^11^C]WAY-100635 for clinical PET applications. Appl Rad Isot.

[CR60] Kreisl WC, Lyoo CH, Liow JS, Wei M, Snow J, Page E, Jenko KJ, Morse CL, Zoghbi SS, Pike VW, Turner RS, Innis RB (2016). ^11^C-PBR28 binding to translocator protein increases with progression of Alzheimer’s disease. Neurobiol Aging.

[CR61] Kuhl DE, Koeppe RA, Minoshima S, Snyder SE, Ficaro EP, Foster NL, Frey KA, Kilbourn MR (1999). In vivo mapping of cerebral acetylcholinesterase activity in aging and Alzheimer’s disease. Neurology.

[CR62] Kuhl DE, Koeppe RA, Snyder SE, Minoshima S, Frey KA, Kilbourn MR (2006). In vivo butyrylcholinesterase activity is not increased in Alzheimer’s disease synapses. Ann Neurol.

[CR63] Laymon CM, Narendran R, Mason NS, Carney JP, Lopresti BJ, Mathis CA, Mountz JM, Sashin D, Frankle WG (2012). Human biodistribution and dosimetry of the PET radioligand [^11^C]flumazenil (FMZ). Mol Imaging Biol.

[CR64] Lin K-J, Weng Y-H, Wey S-P, Hsiao I-T, Lu C-S, Skovronsky D, Chang H-P, Kung M-P, Yen T-C (2010). Whole-body biodistribution and radiation dosimetry of ^18^F-FP-(+)-DTBZ (^18^F-AV-133): a novel vesicular monoamine transporter 2 imaging agent. J Nucl Med.

[CR65] Lu J-Q, Ichise M, Liow J-S, Ghose S, Vines D, Innis RB (2004). Biodistribution and radiation dosimetry of the serotonin transporter ligand ^11^C-DASB determined from human whole-body PET. J Nucl Med.

[CR66] McParland BJ, Lax M, Axelsson J, Wall A, Johansson S, Sorensen J (2010). The biodistribution and internal radiation dosimetry of [^18^F]GE-148 in healthy adult volunteers. Eur J Nucl Med Mol Imaging.

[CR67] Mejia AA, Nakamura T, Itoh M, Hatazawa J, Ishiwata K, Ido T, Matsumoto M, Watabe H, Watanuki S, Seo S (1991). Absorbed dose estimated in positron emission tomography studies based on the administration of ^18^F-labeled radiopharmaceuticals. J Radiat Res.

[CR68] Mendes BM, Ferreira AV, Nascimento LTC, Ferreira SMZMD, Silveira FMB, Silva JB (2018). New radiation dosimetry estimates for [^18^F]FLT based on voxelized phantoms. Rad Res.

[CR69] Mickey BJ, Ducci F, Hadgkinson CA, Langenecker SA, Goldman D, Zubieta J-K (2008). Monoamine oxidase A genotype predicts human serotonin 1A receptor availability in vivo. J Neurosci.

[CR70] Miller S, Li P, Schipper M, Junck L, Piert M, Lawrence TS, Tsien C, Cao Y, Kim MM (2019). Metabolic tumor volume response assessment using ^11^C-methionine positron emission tomography identifies glioblastoma tumor subregions that predict progression better than baseline or anatomic magnetic resonance imaging alone. Adv Radiat Oncol.

[CR71] Minn H, Kauhanen S, Seppänen M, Nuutila P (2009). ^18^F-FDOPA: a multiple-target molecule. J Nucl Med.

[CR72] Minoshima S, Koeppe RA, Fessler JA, Mintun MA, Berger KL, Taylor SF, Kuhl DE, Ue-Mura K, Lassen NA, Jones T, Kanno I (1993). Integrated and automated data analysis method for neuronal activation studies using O-15 water PET. Quantification of brain function: tracer kinetics and image analysis in brain PET, International congress series 1030.

[CR73] Moran M, Wilson A, Stableford W, Wong M, Houle S, Vasdev N (2010). A one-step radiosynthesis of [^11^C]methylphenidate. J Nucl Med.

[CR74] Mosessian S, Duarte-Vogel SM, Stout DB, Roos KP, Lawson GW, Jordan MC, Ogden A, Matter C, Sadeghi S, Mills GQ, Schelbert HR, Radu CG, Czernin J, Couto M, Phelps ME (2014). INDs for PET molecular imaging probes-approach by an academic institution. Mol Imaging Biol.

[CR75] Mossine AV, Brooks AF, Henderson BD, Hockley BG, Frey KA, Scott PJH (2017). An updated radiosynthesis of [^18^F]AV1451 for Tau PET imaging. EJNMMI Radiopharm Chem.

[CR76] Mossine AV, Tanzey SS, Brooks AF, Makaravage KJ, Ichiishi N, Miller JM, Henderson BD, Skaddan M, Sanford MS, Scott PJH (2019). One-pot synthesis of high molar activity 6-[^18^F]Fluoro-L-DOPA by cu-mediated fluorination of a BPin precursor org. Biomol Chem.

[CR77] Mossine AV, Tanzey SS, Brooks AF, Makaravage KJ, Ichiishi N, Miller JM, Henderson BD, Skaddan MB, Sanford MS, Scott PJH (2020). Synthesis of high molar activity 6-[^18^F]Fluoro-L-DOPA suitable for human use by cu-mediated fluorination of a BPin precursor. Nat Prot.

[CR78] Mulholland GK, Jewtt DM, Toorongian SA (1988). Routine synthesis of N-[^11^C-methyl]scopolamine by phosphite mediated reductive methylation with [^11^C]formaldehyde. Appl Radiat Isot.

[CR79] Mulholland GK, Kilbourn MR, Sherman P, Carey JE, Frey KA, Koeppe RA, Kuhl DE (1995). Synthesis, in vivo biodistribution and dosimetry of [^11^C]N-Methylpiperidyl Benzilate ([^11^C]NMPB), a muscarinic acetylcholine receptor antagonist. Nucl Med Biol.

[CR80] Mulholland GK, Otto CA, Jewett DM, Kilbourn MR, Koeppe RA, Sherman PS, Petry NA, Carey JE, Atkinson ER, Archer S, Frey KA, Kuhl DE (1992). Synthesis, rodent biodistribution, dosimetry, metabolism, and monkey images of carbon-11- labeled (+)-2α-Tropanyl Benzilate: a central muscarinic receptor imaging agent. J NucI Med.

[CR81] Münch G, Nguyen NT, Nekolla S, Ziegler S, Muzik O, Chakraborty P, Wieland DM, Schwaiger M (2000). Evaluation of sympathetic nerve terminals with [^11^C]epinephrine and [^11^C]hydroxyephedrine and positron emission tomography. Circulation.

[CR82] Murthy R, Harris P, Simpson N, Van Heertum R, Leibel R, Mann JJ, Parsey R (2008). Whole body [^11^C]-dihydrotetrabenazine imaging of baboons: biodistribution and human radiation dosimetry estimates. Eur J Nucl Med Mol Imaging.

[CR83] Naganawa M, Zheng M-Q, Henry S, Nabulsi N, Lin S-F, Ropchan J, Labaree D, Najafzadeh S, Kapinos M, Tauscher J, Neumeister A, Carson RE, Huang Y (2015). Test-retest reproducibility of binding parameters in humans with ^11^C-LY2795050, an antagonist PET radiotracer for the kappa opioid receptor. J Nucl Med.

[CR84] NETSPOT Prescribing Information. 2016. https://www.accessdata.fda.gov/drugsatfda_docs/label/2016/208547s000lbl.pdf. Accessed 7 Jul 2020.

[CR85] Newberg AB, Ray R, Scheuermann J, Wintering N, Saffer J, Schmitz A, Freifelder R, Karp J, Lerman C, Divgi C (2009). Dosimetry of ^11^C-carfentanil, a μ-opioid receptor imaging agent. Nucl Med Commun.

[CR86] Nye JA, Schuster DM, Yu W, Camp VM, Goodman MM, Votaw JR (2007). Biodistribution and radiation dosimetry of the synthetic nonmetabolized amino acid analogue anti-^18^F-FACBC in humans. J Nucl Med.

[CR87] O’Keefe GJ, Saunder TH, Ng S, Ackerman U, Tochon-Danguy HJ, Chan JG, Gong S, Dyrks T, Lindemann S, Holl G, Dinkelborg L, Villemagne V, Rowe CC (2009). Radiation dosimetry of b-amyloid tracers ^11^C-PiB and ^18^F-BAY94-9172. J Nucl Med.

[CR88] Parsey RV, Belanger MJ, Sullivan GM, Simpson NR, Stabin MG, Van Heertum R, Mann JJ (2005). Biodistribution and radiation dosimetry of ^11^C-WAY100,635 in humans. J Nucl Med.

[CR89] Petrou M, Frey KA, Kilbourn MR, Scott PJH, Raffel DM, Bohnen NI, Müller MLTM, Albin RL, Koeppe RA (2014). In vivo imaging of human cholinergic nerve terminals with (−)-5-^18^F-Fluoroethoxybenzovesamicol: biodistribution, dosimetry, and tracer kinetic analyses. J Nucl Med.

[CR90] Piert M, Park H, Khan A, Siddiqui J, Hussain H, Chenevert T, Wood D, Johnson T, Shah RB, Meyer C (2009). Detection of aggressive primary prostate cancer with ^11^C-choline PET/CT using multimodality fusion techniques. J Nucl Med.

[CR91] Piert M, Shao X, Raffel DM, Davenport M, Montgomery J, Kunju P, Hockley BG, Siddiqui J, Scott PJH, Chinnaiyan AM, Rajendiran T (2017). Preclinical evaluation of 11C-sarcosine as a substrate of proton-coupled amino acid transporters and first human application in prostate cancer. J Nucl Med.

[CR92] Quarles RP, Mintun MA, Larson KB, Markham J, MacLeod AM, Raichle ME (1993). Measurement of regional cerebral blood flow with positron emission tomography: a comparison of [^15^O]water to [^11^C]Butanol with distributed-parameter and compartmental models. J Cerebral Blood Flow Metab.

[CR93] Raffel DM, Corbett JR, Del Rosario RB, Gildersleeve DL, Chiao P-C, Schwaiger M, Wieland DM (1996). Clinical evaluation of carbon-11-phenylephrine: MAO-sensitive marker of cardiac sympathetic neurons. J Nucl Med.

[CR94] Raffel DM, Jung Y-W, Koeppe RA, Jang KS, Gu G, Scott PJH, Murthy VL, Rothley J, Frey KA (2018). First-in-human studies of [^18^F]Fluorohydroxyphenethylguanidines: PET radiotracers for quantifying regional cardiac sympathetic nerve density. Circ Cardiovasc Imaging.

[CR95] Ribeiro M-J, Ricard M, Bourgeois S, Liévre M-A, Bottlaender M, Gervais P, Dollé F, Syrota A (2005). Biodistribution and radiation dosimetry of [^11^C]raclopride in healthy volunteers. Eur J Nucl Med Mol Imaging.

[CR96] Ribeiro M-J, Ricard M, Liévre M-A, Bourgeois S, Edmond P, Gervais P, Dollé F, Syrota A (2007). Whole-body distribution and radiation dosimetry of the dopamine transporter radioligand [^11^C]PE2I in healthy volunteers. Nucl Med Biol.

[CR97] Richards ML, Scott PJH, Hockley BG, Scott PJH (2012). Synthesis of [^18^F]Fluorodeoxyglucose ([^18^F]FDG). Radiochemical syntheses volume 1: radiopharmaceuticals for positron emission tomography.

[CR98] Riss PJ, Ferrari V, Bielik R, Canales-Candela R, Smith R, Aigbirhio FI, Hockley BG, Scott PJH (2012). Synthesis of [^18^F]Fluoromisonidazole (1-(2-Hydroxy-3-[18F]Fluoropropyl)-2-Nitroimidazole, [^18^F]FMISO). Radiochemical syntheses volume 1: radiopharmaceuticals for positron emission tomography.

[CR99] Rodnick ME, Brooks AF, Hockley BG, Henderson BD, Scott PJH (2013). A fully-automated one-pot synthesis of [^18^F]fluoromethylcholine with reduced dimethylaminoethanol contamination via [^18^F]fluoromethyl tosylate. Appl Radiat Isot.

[CR100] Rodnick ME, Sollert C, Stark D, Clark M, Katsifis A, Hockley BG, Parr DC, Frigell J, Henderson BD, Abghari-Gerst M, Piert MR, Fulham MJ, Eberl S, Ggnon K, Scott PJH. Cyclotron-based production of ^68^Ga, [^68^Ga]GaCl_3_, and [^68^Ga]Ga-PSMA-11 from a Liquid target. EJNMMI Radiopharm Chem. 2020, Accepted (Preprint available here: https://www.researchsquare.com/article/rs-38981/v1. 10.21203/rs.3.rs-38981/v1. 10.1186/s41181-020-00106-9 PMC7661618 33180205

[CR101] Runkle AC, Shao X, Tluczek LJ, Henderson BD, Hockley BG, Scott PJH (2011). Automated production of [^11^C] acetate and [^11^C]palmitate using a modified GE Tracerlab FX_C-Pro_. Appl Radiat Isot.

[CR102] Sandgren K, Johansson L, Axelsson J, Jonsson J, Ögren M, Ögren M, Andersson M, Strandberg S, Nyholm T, Riklund K, Widmark A (2019). Radiation dosimetry of [^68^Ga]PSMA-11 in low-risk prostate cancer patients. EJNMMI Phys.

[CR103] Sattler B, Kranz M, Patt M, Donat C, Deuther-Conrad W, Hoepping A, Sattler T, Steinbach J, Brust P, Sabri O (2012). Incorporation dosimetry of F-18-Flubatine - comparison of animal model data with first-in-man results. J Nucl Med.

[CR104] Savi A, Incerti E, Fallanca F, Bettinardi V, Rosseti F, Monterisi C, Compierchio A, Negri G, Zannini P, Gianolli L, Picchio M (2017). First evaluation of PET-based human biodistribution and dosimetry of ^18^F-FAZA, a tracer for imaging tumor hypoxia. J Nucl Med.

[CR105] Schwarz SW, Decristoforo C (2019). US and EU radiopharmaceutical diagnostic and therapeutic nonclinical study requirements for clinical trials authorizations and marketing authorizations. EJNMMI Radiopharm Chem.

[CR106] Schwarz SW, Decristoforo C, Goodbody AE, Singhal N, Saliba S, Ruddock PS, Zukotynski K, Ross AA (2019). Harmonization of U.S., European Union, and Canadian first-in-human regulatory requirements for radiopharmaceuticals: is this possible?. J Nucl Med.

[CR107] Schwarz SW, Dick D, VanBrocklin HF, Hoffman JM (2014). Regulatory requirements for PET drug production. J Nucl Med.

[CR108] Scott DJ, Heitzeg MM, Koeppe RA, Stohler CS, Zubieta J-K (2006). Variations in the human pain stress experience mediated by ventral and dorsal basal ganglia dopamine activity. J Neurosci.

[CR109] Scott PJH, Hockley BG, Scott PJH (2012). Synthesis of [^13^N]Ammonia. Radiochemical syntheses volume 1: radiopharmaceuticals for positron emission tomography.

[CR110] Segall G, Delbeke D, Stabon MG, Even-Sapir E, Fair J, Sajdak R, Smith GT (2010). SNM practice guideline for sodium ^18^F-fluoride PET/CT bone scans 1.0. J Nucl Med.

[CR111] Seltzer MA, Jahan SA, Sparks R, Stout DB, Satyamurthy N, Dahlbom M, Phelps ME, Barrior JR (2004). Radiation dose estimates in humans for ^11^C-acetate whole-body PET. J Nucl Med.

[CR112] Shao X, Fawaz MV, Jang K, Scott PJH (2014). Ethanolic carbon-11 chemistry: the introduction of green radiochemistry. Appl Radiat Isot.

[CR113] Shao X, Hoareau R, Hockley BG, Tluczek LJ, Henderson BD, Padgett HC, Scott PJHSA (2011). Highlighting the versatility of the Tracerlab synthesis modules. Part 1: fully automated production of [^18^F]Labelled radiopharmaceuticals using a Tracerlab FX_FN_. J Labelled Comp Radiopharm.

[CR114] Shao X, Hoareau R, Runkle AC, Tluczek LJM, Hockley BG, Henderson BD, Scott PJH (2011). Highlighting versatility of the Tracerlab synthesis modules. Part 2: fully automated production of [^11^C]-labeled radiopharmaceuticals using a Tracerlab FX_C-Pro_. J Labelled Comp Radiopharm.

[CR115] Shao X, Jang K, Scott PJH, Scott PJH (2015). Synthesis of 1-(2,4-Dichlorophenyl)-4-cyano-5-(4- [^11^C]methoxyphenyl)-N-(piperidin-1-yl)-1H-pyrazole-3-carboxamide ([^11^C]OMAR). Radiochemical syntheses volume 2: further radiopharmaceuticals for positron emission tomography and new strategies for their production.

[CR116] Silveira MB, Soares MA, Valente ES, Waquil SS, Ferreira AV, dos Santost RG, da Silva JB (2010). Synthesis, quality control and dosimetry of the radiopharmaceutical ^18^F-sodium fluoride produced at the Center for Development of Nuclear Technology – CDTN. Braz J Pharm Sci.

[CR117] Snellman A, Rokka J, López-Picón FR, Eskola O, Salmona M, Forloni G, Scheinin M, Solin O, Rinne JO, Haaparanta-Solin M (2014). In vivo PET imaging of beta-amyloid deposition in mouse models of Alzheimer’s disease with a high specific activity PET imaging agent [^18^F]flutemetamol. EJNMMI Res.

[CR118] Snyder SE, Gunupudi N, Sherman PS, Butch ER, Skaddan MB, Kilbourn MR, Koeppe RA, Kuhl DE (2001). Radiolabeled cholinesterase substrates: in vitro methods for determining structure-activity relationships and identification of a positron emission tomography radiopharmaceutical for in vivo measurement of butyrylcholinesterase activity. J Cereb Blood Flow Metab.

[CR119] Songmen S, Nepal P, Olsavsky T, Sapire J (2019). Axumin positron emission tomography: novel agent for prostate cancer biochemical recurrence. J Clin Imaging Sci.

[CR120] Sörensen J, Owenius R, Lax M, Johansson S (2013). Regional distribution and kinetics of [^18^F]fluciclovine (anti-[^18^F]FACBC), a tracer of amino acid transport, in subjects with primary prostate cancer. Eur J Nucl Med Mol Imaging.

[CR121] Sowa AR, Jackson IM, Desmond TJ, Alicea J, Mufarreh AJ, Pham JM, Stauff J, Winton WP, Fawaz MV, Henderson BD, Hockley BG, Rogers VE, Koeppe RA, Scott PJH (2018). Futureproofing [^18^F]Fludeoxyglucose manufacture at an Academic Medical Center. EJNMMI Radiopharm Chem.

[CR122] Srinivasan S, Crandall JP, Gajwani P, Sgouros G, Mena E, Lodge MA, Wahl RL (2020). Human radiation dosimetry for orally and intravenously administered ^18^F-FDG. J Nucl Med.

[CR123] Stabin MG, Sparks RB, Crowe E (2005). The second-generation personal computer software for internal dose assessment in nuclear medicine. J Nucl Med.

[CR124] Suleiman OH, Fejka R, Houn F, Walsh M (2006). The radioactive drug research committee: background and retrospective study ofm reported research data. J Nucl Med.

[CR125] Tolvanen T, Yli-Kerttula T, Ujula T, Autio A, Lehikoinen P, Minn H, Roivainen A (2010). Biodistribution and radiation dosimetry of [^11^C]choline: a comparison between rat and human data. Eur J Nucl Med Mol Imaging.

[CR126] USP <823> Positron Emission Tomography Drugs for Compounding, Investigational, and Research Uses (2020). Radiopharmaceuticals for positron emission tomography. USP 43-NF 38.

[CR127] VanBrocklin HF (2008). Radiopharmaceuticals for drug development: United States regulatory perspective. Curr Radiopharm.

[CR128] Vander Borght TM, Kilbourn MR, Koeppe RA, DaSilva JN, Carey JE, Kuhl DE, Frey KA (1995). In vivo imaging of the brain vesicular monoamine transporter. J NucI Med.

[CR129] Vesselle H, Grierson J, Peterson LM, Muzi M, Mankoff DA, Krohn KA (2003). ^18^F-Fluorothymidine radiation dosimetry in human PET imaging studies. J Nucl Med.

[CR130] Virta JR, Tolvanen T, Någren K, Brück A, Roivainen A, Rinne JO (2008). 1-^11^C-Methyl-4-piperidinyl-N-butyrate radiation dosimetry in humans by dynamic organ-specific evaluation. J Nucl Med.

[CR131] Wagner P (2005). US EPA, Inert Reassessment – n-Butanol, CAS # 71–36-3 and Isobutyl Alcohol, CAS # 78–83-1.

[CR132] Walker RC, Smith GT, Liu E, Moore B, Clanton J, Stabin M (2013). Measured human dosimetry of ^68^Ga-DOTATATE. J Nucl Med.

[CR133] Washburn LC, Sun TT, Byrd BL, Rafter JJ, Hayes RL, Frey KA, Agranoff BW, Raynaud C (1982). ^11^C-ACHC, a potential agent for positron tomographic measurement of brain amino acid transport. Nuclear medicine and biology: proceedings of the third world congress of nuclear medicine and biology, August 29–September 2, 1982, Paris, France.

[CR134] Watkins GL, Jewett DM, Mulholland GK, Kilbourn MR, Toorongian SA (1988). A captive solvent method for rapid N-[^11^C]methylation of secondary amides: application to the benzodiazepine, 4′-Chlorodiazepam (RO5-4864). Appl Radiat Isot.

[CR135] Wong DF, Kuwabara H, Horti AG, Raymont V, Brasic J, Guevara M, Ye W, Dannals RF, Ravert HT, Nandi A, Rahmim A, Ming JE, Grachev I, Roy C, Cascella N (2010). Quantification of cerebral cannabinoid receptors subtype 1 (CB1) in healthy subjects and schizophrenia by the novel PET radioligand [^11^C]OMAR. Neuroimage.

[CR136] Wong DF, Kuwabara H, Pomper M, Holt DP, Brasic JR, George N, Frolov B, Willis W, Gao Y, Valentine H, Nandi A, Gapasin L, Dannals RF, Horti AG (2014). Human brain imaging of α7 nAChR with [^18^F]ASEM: a new PET radiotracer for neuropsychiatry and determination of drug occupancy. Mol Imaging Biol.

[CR137] Wong KK, Piert M (2013). Dynamic bone imaging with ^99m^Tc-labeled Diphosphonates and ^18^F-NaF: mechanisms and applications. J Nucl Med.

[CR138] Wrobel MC, Carey JE, Sherman PS, Kilbourn MR (1997). Simplifying the dosimetry of carbon-11-labeled radiopharmaceuticals. J Nucl Med.

[CR139] Yang L, Brooks AF, Makaravage KJ, Zhang H, Sanford MS, Scott PJH, Shao X (2018). Radiosynthesis of [^11^C]LY2795050 for preclinical and clinical PET imaging using cu(II)-mediated Cyanation. ACS Med Chem Lett.

[CR140] Yi C, Yu D, Shi X, He Q, Zhang X, Zhang X (2015). Biodistribution and estimation of radiation-absorbed doses in humans for ^13^N-ammonia PET. Ann Nucl Med.

[CR141] Yoshida T, Kuwabara Y, Ichiya Y, Sasaki M, Fukumura T, Ichimiya A, Takita M, Ogomori K, Masuda K (1998). Cerebral muscarinic acetylcholinergic receptor measurement in Alzheimer's disease patients on ^11^C-N-methyl-4-piperidyl benzilate — comparison with cerebral blood flow and cerebral glucose metabolism. Ann Neurol.

[CR142] Zubieta JK, Greenwald MK, Lombardi U, Woods JH, Kilbourn MR, Jewett DM, Koeppe RA, Schuster CR, Johanson CE (2000). Buprenorphine-induced changes in mu-opioid receptor availability in male heroin-dependent volunteers: a preliminary study. Neuropsychopharmacology.

[CR143] Zubieta JK, Koeppe RA, Frey KA, Kilbourn MR, Mangner TJ, Foster NL, Kuhl DE (2001). Assessment of muscarinic receptor concentrations in aging and Alzheimer disease with [^11^C]NMPB and PET. Synapse.

