# Supplementary material for: Use of 55 PET radiotracers under approval of a Radioactive Drug Research Committee (RDRC)
Source: EJNMMI Radiopharm Chem. 2020 Nov 11;5:24. doi: 10.1186/s41181-020-00110-z (PMC7658275; doi:10.1186/s41181-020-00110-z)
Supplement: Supplementary file 1 — Additional file 1. [file 41181_2020_110_MOESM1_ESM.docx]

**Use of 55 PET Drugs under Approval of a Radioactive Drug Research**

**Committee (RDRC)**

Isaac M. Jackson,^a,b^ So Jeong Lee,^a,b^ Alexandra R. Sowa,^a^ Melissa E. Rodnick,^a^

Laura Bruton,^a^ Mara Clark,^a^ Sean Preshlock,^a^ Jill Rothley,^a^ Virginia E. Rogers,^a^

Leslie E. Botti,^a^ Bradford D. Henderson,^a^ Brian G. Hockley,^a^ Jovany Torres,^a^

David M. Raffel,^a^ Allen F. Brooks,^a^ Kirk A. Frey,^a^ Michael R. Kilbourn,^a^

Robert A. Koeppe,^a^ Xia Shao,^a^ Peter J. H. Scott^a,^*

1. Department of Radiology, University of Michigan, Ann Arbor MI, USA.
2. Current addresses. IMJ: Stanford University, Stanford CA, USA; SJL: Gordon Center for Medical Imaging, Massachusetts General Hospital, Harvard Medical School, Boston MA, USA.

* Correspondence: [pjhscott@umich.edu](mailto:pjhscott@umich.edu)

**Table of Contents**

| **1.** | **Radiosyntheses used at UM and not previously published** | S2 |
| --- | --- | --- |
| 1.1 | [^11^C]Butanol | S2 |
| 1.2 | [^18^F]ASEM | S3 |
| 1.3 | [^18^F]FDOPA | S4 |
| 1.4 | [^68^Ga]Ga-PSMA-11 | S5 |
| **2.** | **Quality control testing** | S7 |
| **3.** | **Dosimetry not previously published** | S10 |
| 3.1 | [^18^F]ASEM Dosimetry | S10 |
| 3.2 | [^11^C]Butanol Dosimetry | S11 |
| 3.3 | [^11^C]HED Dosimetry | S12 |
| 3.4 | [^11^C]LY2795050 | S13 |
| 3.5 | [^11^C]MPH Dosimetry | S14 |
| 3.6 | [^18^F]MPPF Dosimetry | S15 |
| 3.7 | [^11^C]PMP Dosimetry | S16 |
| 3.8 | [^11^C]Ro-54864 Biodistribution | S17 |
| **4.** | **Rationale for no mass dose limit in certain cases** | S18 |
| **5.** | **References** | S20 |

1. **Radiosyntheses used at UM and not previously published**

**1.1 [^11^C]Butanol**

**Scheme S1.** Radiosynthesis of [^11^C]butanol

*Radiosynthesis:* [^11^C]butanol was prepared in a TRACERlab FX_C-Pro_ synthesis module modified as previously reported (Mossine *et al*., 2016). The synthesis module was loaded as follows: reaction vessel: n-propyl magnesium chloride in ether (50 μL of 2 M solution diluted with 950 μL ether); Vial 1: lithium aluminum hydride (LAH) (150 μL of 1 M solution diluted into 850 μL ether); Vial 2: HCl (1.5 mL of 1 M aqueous solution); Vial 3: Water (10 mL); Vial 4: Water (5 mL); Vial 5: Ethanol (0.5 mL); Vial 6: Sterile saline for injection (2.5 mL); round-bottomed dilution flask: water (10 mL); product vial: Sterile saline for injection (7 mL). [^11^C]CO_2_ (~111GBq) was bubbled into the Grignard solution at 15 mL/min for 4 minutes. LAH solution was then transferred in to the reaction mixture. After 1 minute, ether was evaporated under He stream at 120 mL/min for 5 minutes at room temperature. HCl was added, and then the crude reaction mixture was transferred to the dilution flask containing 10 mL water, followed by 5 mL water to rinse the reactor. This mixture was passed through the Waters C18 Plus Long cartridge. The cartridge was washed with 5 mL water to remove impurities from the cartridge, and dried for 1.0 min with He gas. [^11^C]Butanol was eluted with 0.5 mL EtOH and 2.5 mL USP saline into the product vial containing 7 mL saline. The product was then passed through a Millipore-GS 0.22-μm filter into a sterile 10 mL dose vial (Hollister-Stier), and submitted for QC testing. Total synthesis time was 35 minutes from end of beam. Typically 5.1 ± 0.8 GBq (4.6 ± 0.7 % non-decay-corrected RCY) of [^11^C]butanol were obtained from approximately 111 GBq of

[^11^C]CO_2_ (n = 3).

*Quality control.* Testing was completed according to our standard QC methods (Section 2) and results for three validation batches are provided (Table S1).

**Table S1:** Synthesis Data for [^11^C]Butanol.

| QC Test | Release criteria | Batch 1 | Batch 2 | Batch 3 |
| --- | --- | --- | --- | --- |
| Visual inspection | Clear, no ppt | Pass | Pass | Pass |
| pH | 4.5-7.5 | 5.5 | 5.5 | 5.0 |
| Radioactivity Conc. | ≥ 1.1 GBq/10 mL | 4.6 | 4.7 | 6.1 |
| Radiochemical Purity | ≥ 90% | 100 | 100 | 100 |
| Radiochemical Identity | 0.9 - 1.1 | 1.03 | 1.04 | 1.04 |
| Radionuclidic Identity | T_1/2_=18.2-22.4 | 20.3 | 20.2 | 20.2 |
| Filter Integrity | ≥ 44 psi | 48 | 49 | 50 |
| Endotoxin | ≤ 17.5 EU/mL | < 2 | < 2 | < 2 |
| Sterility | Sterile | Sterile | Sterile | Sterile |

**1.2 [^18^F]ASEM**

***Scheme S2****: Synthesis of [^18^F]ASEM*

*Radiosynthesis:* [^18^F]ASEM was prepared in a TRACERlab FX_FN_ synthesis module through adaptation of published methods (Gao *et al*., 2013). The synthesis module was loaded as follows: reaction vessel: Vial 1: K_2_CO_3_ (3.5 mg in 0.5 mL milli-Q water); Vial 2: kryptofix-2.2.2 (15 mg in 1 mL acetonitrile); Vial 3: nitro-precursor (1.0 mg in 1 mL DMF, see Scheme S2); Vial 6: HPLC mobile phase (3 mL); Vial 7: USP saline (9.5 mL), Vial 8: Ethanol, USP (0.5 mL); Vial 9: Milli-Q water (10 mL); round-bottomed dilution flask: water (60 mL) + 1 M NaOH (2.5 mL). [^18^F]Fluoride (approx. 66.6 GBq) was delivered from a PETTrace cyclotron and trapped on a Chromafix PSCO_3_ Sep-Pak cartridge (Waters) that had been preconditioned with KHCO_3_ solution (0.5 M). [^18^F]Fluoride was eluted with a solution of K_2_CO_3_ (3.5 mg) in water (0.5 mL). A solution of kryptofix-2.2.2(15 mg) in acetonitrile (1 mL) was then added to the reactor, and the [^18^F]fluoride was azeotropically dried. A solution of precursor (1.0 mg) in anhydrous DMF (1.0 mL) was added and the solution was heated at 120 °C for 10 min. After cooling to 50 °C, the reaction mixture was diluted with HPLC mobile phase (3 mL) and purified by semi-preparative HPLC (column: Phenomenex Luna C18(2) 250 × 10 mm 10µ; mobile phase: 75% water : 25% acetonitrile : 0.05% TFA (v/v); flow rate: 4.0 mL/min). The fraction corresponding to [^18^F]ASEM (typically eluting around 27 – 29 min) was collected and transferred into a dilution flask containing sterile water (60 mL) and 1M NaOH (2.5 mL). The product was reformulated from the resulting solution by trapping on a 1 cc C18 cartridge (Waters), rinsing with sterile water (10 mL), eluting into a second collection vial with ethanol for injection (0.5 mL) and diluting with 0.9% sodium chloride for injection (9.5 mL). The final isotonic formulation (10 mL) was passed through a 0.22 μM Millex-GV sterile filter (Millipore) into a sterile 10 mL dose vial (Hollister-Stier) to provide [^18^F]ASEM. Typically 7.8 ± 2.1 GBq (11.7 ± 3.0% non-decay-corrected RCY) of [^18^F]ASEM were obtained from approximately 66.6 GBq of [^18^F]fluoride (n=4).

*Quality control.* Testing was completed according to our standard QC methods (Section 2) and results for three validation batches are provided (Table S2).

**Table S2:** Synthesis data for [^18^F]ASEM

| QC Test | Release criteria | Batch 1 | Batch 2 | Batch 3 | Batch 4 |
| --- | --- | --- | --- | --- | --- |
| Visual inspection | Clear, no ppt | Pass | Pass | Pass | Pass |
| pH | 4.5-7.5 | 5.0 | 5.0 | 7.1 | 5.0 |
| Radiochemical purity | ≥90% | 96 | 98 | 98 | 97 |
| Radioactivity Conc. | ≥ 0.037 GBq/10 mL | 11.0 | 6.8 | 7.1 | 6.3 |
| ASEM Conc. | Report µg/mL | 2.7 | 1.0 | 1.3 | ND |
| Molar Activity | A_m_ GBq/µmol | 146 | 247 | 192 | 195 |
| Radiochemical Identity | RRT = 0.9 - 1.1 | 1.0 | 1.0 | 1.0 | 1.0 |
| Residual K_2.2.2_ | ≤50 µg | Pass | Pass | Pass | Pass |
| Radionuclidic Identity | T_1/2_=105-115 min | 108 | 108 | 107 | 111 |
| Residual Solvent Analysis | ≤410 ppm MeCN | 16 | 9 | 20 | ND |
|  | ≤880 ppm DMF | ND | ND | 6 | ND |
| Filter Integrity | ≥ 44 psi | 52 | 55 | 51 | 52 |
| Endotoxin | ≤ 17.5 EU/mL | < 2 | < 2 | < 2 | <2 |
| Sterility | Sterile | Sterile | Sterile | Sterile | Sterile |

RRT = Relative retention time (t_R_ [^18^F]ASEM/t_R_ ASEM Std); ND = not detected.

**1.3 [^18^F]FDOPA**

[^18^F]FDOPA can be prepared via our previously reported copper-mediated radiofluorination of a pinacol boronate ester (Mossine *et al*., 2019, 2020), or via the traditional nucleophilic aromatic substitution approach as shown in Scheme S3 and outlined below.

**

***Scheme S3****: Synthesis of [^18^F]FDOPA*

*Radiosynthesis:* [^18^F]FDOPA was prepared in a FASTLab2 synthesis module using a commercially available kit (ABX pn DP-02-H) and reagents (ABX pn DP-102). [^18^F]Fluoride (approx. 66.6 GBq) was delivered from a PETTrace cyclotron and trapped on a QMA Sep-Pak. The trapped [^18^F]fluoride was then eluted to the reaction vessel using the tetrabutyl-ammonium hydrogen carbonate solution (1.05 mL, 0.075 M). The subsequent azeotropic drying steps with anhydrous acetonitrile provided the reactive fluorinating reagent. FDOPA precursor (30.0 mg dissolved in 1.3 mL anhydrous DMSO, see Scheme S3) was then added to the reaction vessel and the initial radiofluorination occurred at 130 ^o^C for 8 minutes. Following fluorination, the crude intermediate was isolated via solid-phase extraction using a C18 end capped (C18ec) cartridge. This initial intermediate was then transferred to the reaction vessel, followed by addition of mCPBA (18.0 mg), and the oxidation process occurred in acetonitrile (65 ^o^C, 15 min). Ethanol (1.3 mL) and 30% hydrochloric acid (2.1 mL) were then added to the crude reaction mixture and the acidic de-protection was carried out at 40 ^o^C for 20 min. Following the completion of the acidic hydrolysis step, the final product was purified via further solid-phase extraction with C18 and H-RP cartridges. The final product solution was then passed through anion exchange (WAX) and alumina cartridges to remove any traces of unreacted [^18^F]fluoride, before being sterile-filtered (Millipore Cathivex-GV 0.22 µm) into a sterile 30 mL dose vial (Hollister-Stier) to yield formulated [^18^F]FDOPA in 27-28 mL of phosphate buffer containing 3% ethanol. Typically 5.0 ± 0.6 GBq (7.5 ± 0.9% non-decay-corrected RCY) of [^18^F]FDOPA were obtained from approximately 66.6 GBq of [^18^F]fluoride (n=4).

*Quality control.* Testing was completed according to our standard QC methods (Section 2) and results for four validation batches are provided (Table S3).

**Table S3:** Synthesis data for [^18^F]FDOPA

| QC Test | Release criteria | Batch 1 | Batch 2 | Batch 3 | Batch 4 |
| --- | --- | --- | --- | --- | --- |
| Visual inspection | Clear, no ppt | Pass | Pass | Pass | Pass |
| pH | 4.5-7.5 | 5.0 | 5.5 | 5.5 | 5.0 |
| Radioactivity Conc. | ≥ 0.037 GBq/28 mL | 5.5 | 4.7 | 5.4 | 4.4 |
| FDOPA Conc. | ≤5 µg/mL | 0.4 | 0.4 | 0.6 | 0.3 |
| Total impurities | ≤50 µg/mL | 4.5 | 5.6 | 4.7 | 4.9 |
| Molar Activity | A_m_ GBq/µmol | 96 | 96 | 67 | 122 |
| Enantiomeric Purity | ≥95% L | 99% | 100 | 100 | 100 |
| Radiochemical Purity | ≥ 90% | 99% | 100 | 100 | 100 |
| Radiochemical Identity | RRT = 0.9 - 1.1 | 1.1 | 1.0 | 1.0 | 1.0 |
| Residual TBA^+^ | ≤100 µg | Pass | Pass | Pass | Pass |
| Radionuclidic Identity | T_1/2_=105-115 min | 107 | 110 | 107 | 107 |
| Residual Solvent Analysis | ≤410 ppm MeCN | 107 | 86 | 83 | ND |
|  | ≤5k ppm DMSO | ND | ND | 80 | 0.1 |
| Filter Integrity | ≥ 44 psi | 51 | 51 | 52 | 51 |
| Endotoxin | ≤ 17.5 EU/mL | < 2 | < 2 | < 2 | <2 |
| Sterility | Sterile | Sterile | Sterile | Sterile | Sterile |

RRT = Relative retention time (t_R_ [^18^F]FDOPA/t_R_ FDOPA Std); ND = not detected.

**1.4 [^68^Ga]Ga-PSMA-11**

[^68^Ga]Ga-PSMA-11 can be prepared from cyclotron-produced gallium-68 using our previously reported method (Rodnick *et al*., 2020), or via the traditional method using generator-derived gallium-68 as shown in Scheme S4 and outlined below.


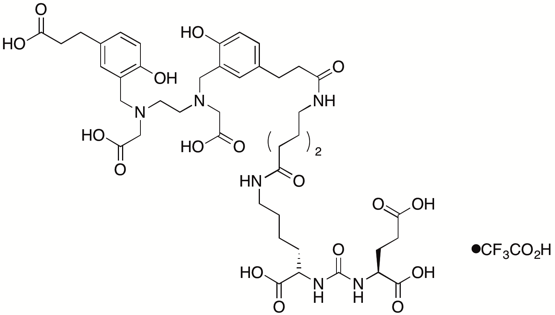

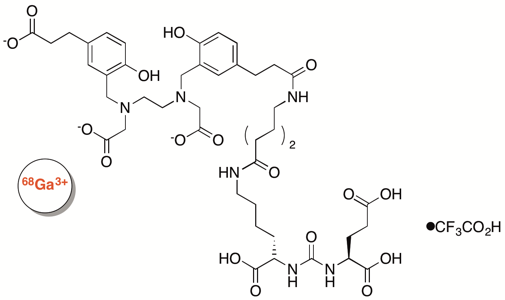


***Scheme S4****: Synthesis of [^68^Ga]Ga-PSMA-11*

*Radiosynthesis:* [^68^Ga]Ga-PSMA-11 was prepared using a Scintomics GRP synthesis module and commercially available kit (ABX Reagent and hardware kit for synthesis of Ga-68 peptides). [^68^Ga]GaCl_3_ (≤1.85 GBq depending upon the age of the generator) was eluted from an itG ^68^Ge/^68^Ga generator with 0.05 M HCl (4 mL). The eluent was diluted with sterile water for injection (9 mL) and the mixture passed through a cation exchange cartridge (PS-H^+^, ABX) to trap [^68^Ga]GaCl_3_. [^68^Ga]GaCl_3_ was then eluted with 5 M NaCl (1.7 mL) into the reactor of the kit pre-charged with PSMA-11 (10 µg in 1.5 mL Hepes buffer). The reactor was heated at 125 ^o^C for 10 min to generate [^68^Ga]Ga-PSMA-11. The reaction was then cooled, and the mixture purified using solid phase extraction. The crude reaction mixture was loaded onto a C18 Sep-Pak light (Waters) (previously conditioned with ethanol (5 mL) and sterile water for injection (38 mL)). The Sep-Pak was washed with 10 mL of water, dried and the product eluted with a 1:1 mixture of ethanol, USP and sterile water for injection, USP (2 mL) through a Millipore-GV 0.22 µm sterilizing filter into a sterile 30 mL vial (Hollister-Stier) and diluted with phosphate buffered saline (14.5 mL). Typically 1.2 ± 0.3 GBq of [^68^Ga]Ga-PSMA-11 were obtained (n=3).

*Quality control.* Testing was completed according to our standard QC methods (Section 2) and results for four validation batches are provided (Table S4).

**Table S4:** Synthesis data for [^68^Ga]Ga-PSMA-11

| QC Test | Release criteria | Batch 1 | Batch 2 | Batch 3 |
| --- | --- | --- | --- | --- |
| Visual inspection | Clear, no ppt | Pass | Pass | Pass |
| pH | 4.0-8.0 | 7.5 | 7.5 | 7.0 |
| Radioactive Conc. | >0.093 GBq/dose | 1.5 | 1.2 | 0.9 |
| Radiochemical Purity | ≥ 90% | 99 | 99 | 98 |
| Radiochemical Identity | RRT = 0.9 - 1.1 | 1.0 | 1.0 | 1.0 |
| Radionuclidic Identity | T_1/2_=64.6-71.4 | 68.1 | 66.3 | 67.0 |
| Filter Integrity | ≥ 50 psi | 48 | 49 | 50 |
| Endotoxin | ≤ 10.9 EU/mL | < 2 | < 2 | < 2 |
| Sterility | Sterile | Sterile | Sterile | Sterile |
| Residual ^68^Ge | <0.01% | 0.005% | 0.005% | 0.003% |

1. **Quality Control Testing**

Quality control of radiotracers is carried out using standard methods defined in the US Pharmacopeia, and as described below:

**Visual inspection**

Doses are examined visually and must be clear, colorless and free of particulate matter.

**Dose pH**

The pH of doses is analyzed by applying a small amount of the dose to pH-indicator strips and determined by visual comparison with the provided scale. Dose pH must be 4.5 – 7.5.

**HPLC analysis**

Radiochemical purity, concentration and molar activity are determined by radio-HPLC. Co-injection of the dose with unlabeled reference standard is performed to confirm compound identity.

***Butanol.*** Column: Phenomenex Rezex RCM-Monosaccharide Ca+, 300x7.8mm; Column temp: 60 ^o^C; Mobile phase: 100% water; Flow rate: 1.4 mL/min; UV: 205 nm; Retention time: ~12 min.

***ASEM.*** Column: Phenomenex Gemini NX-C18, 250x4.6mm; Column temp: 40 ^o^C; Mobile phase: 30% MeCN / 0.05 % TFA; Flow rate: 1.0 mL/min; UV: 225 nm; Retention time: ~6.5 min.

***FDOPA.*** Column: Daicel Crownpak CR(+), 5 µm,4.0 x 150 mm; Column temp: 40˚C; Mobile phase: 20 mM Perchloric acid; Flow Rate: 1.0 mL/min; UV: 283 nm; Retention time: ~4.5 min.

***PSMA-11.*** Column: Phenomenex Luna C18(2) 250x4.6mm; Column temp: 40˚C; Mobile phase A: 0.1% Trifluoroacetic acid in acetonitrile; Mobile phase B: 0.1% Trifluoroacetic acid in water. Gradient: 5%A : 95%B [0 – 10 min]; 40%A : 70%B [10 – 11 min]; 5%A : 95%B [11.1 – 15 min]; Flow rate: 1.0 mL/min; UV: 205 nm; Retention time: ~11 min.

**Radioactive concentration and Radionuclidic identity**

Radioactivity concentration is determined by measuring activity using a Capintec dose calibrator. To determine radionuclidic identity, half-life was calculated using Eq. (1). Calculated half-life must be within ± 5% of the know half-life for a given radionuclide.

T_1/2_ = -ln2(Time Difference / (ln(ending activity/starting activity))) (**1**)

**Residual Solvent Analysis**

Levels of residual solvents in doses containing Class II solvents are analyzed using gas chromatography and flame ionization detection. Limits of residual solvents are based upon the International Conference on Harmonisation of Technical Requirements for Registration of Pharmaceuticals for Human Use guidelines (e.g. MeCN: ≤410 ppm; DMSO: ≤5000 ppm, see Connelly, 2018).

**Residual TBA^+^ and kryptofix-2.2.2 (^18^F-labeled radiotracers only)**

Residual kryptofix-2.2.2 or TBA^+^ levels in doses of ^18^F-labelled radiotracers is analyzed using the established spot tests (Scott and Kilbourn, 2009; Mossine *et al*., 2019, 2020). Strips of plastic-backed silica gel TLC plates saturated with iodoplatinate reagent are spotted with water (negative control), 50 µg/mL K_2.2.2_ or 100 µg/mL TBA^+^ standard (positive control) and the dose. Spots for the three samples are compared and a visual determination of residual in the dose is made. <50 µg/mL K_2.2.2_ and < 2.8 mg/dose TBA^+^ are acceptable.

**Germanium breakthrough (^68^Ga-labeled radiotracers only)**

The ^68^Ga generator is tested for ^68^Ge breakthrough weekly to determine its fitness for use in clinical production during the following week. A small sample of the generator eluate is tested using a multi-channel analyzer for an initial radioactivity profile. The sample is then stored in a shielded environment for 36-72 h to allow the shorter-lived ^68^Ga isotope to decay to near-zero and the sample is tested again. Decay corrected values for the amount of ^68^Ge are determined and used in conjunction with the initial radioactive profile value to determine the relative amount of ^68^Ge released from the generator. The generator is deemed suitable for use if germanium breakthrough is less than 0.01%.

**Sterile filter integrity test**

The sterile filter from dose preparation (with needle still attached) is connected to a nitrogen supply via a regulator. The needle is then submerged in water and the nitrogen pressure gradually increased. If the pressure is raised above the filter acceptance pressure without seeing a stream of bubbles, the filter is considered intact.

**Bacterial endotoxins**

Endotoxin content in radiopharmaceutical doses is analyzed by a Charles River Laboratories EndoSafe^®^ Portable Testing System and according to the US Pharmacopeia. Doses must contain <175 Endotoxin Units (EU).

**Sterility**

Culture tubes of fluid thioglycolate media (FTM) and tryptic soy broth (TSB) are inoculated with samples of doses and incubated (along with positive and negative controls) for 14 days. FTM is used to test for anaerobes, aerobes and microaerophiles whilst TSB is used to test for non-fastidious and fastidious microorganisms. Culture tubes are visually inspected on the 3rd, 7th and 14th days of the test period and compared to the positive and negative standards. Positive standards must show growth (turbidity) in the tubes, and dose/negative controls must have no culture growth after 14 days to be indicative of sterility.

1. **Dosimetry not previously published**

For RDRC studies, the radiation dose to a subject consists of the sum total of all sources of radiation associated with the research protocol, including the PET radiotracer(s), associated x-ray procedures (including CT scans, PET transmission scans etc.) and any follow-up studies. Since associated procedures are highly site, scanner and procedure specific (e.g. is a site using a PET, PET/CT or PET/MRI scanner?), the dosimetry provided in the section is only for the radiotracer dose. Any additional dose resulting from associated or follow up procedures will need to be accounted for in addition to the radiotracer dose.

**3.1 [^18^F]ASEM***

| **Dose:** | **296 MBq / 8.0 mCi** | |
| --- | --- | --- |
| **Organ** | **rad/ mCi** | **rad/ dose** |
| Whole Body | 0.0159 | 0.1272 |
| **Lens of Eye** | **0.0159** | **0.1272** |
| **Red Marrow** | **0.0103** | **0.0824** |
| **Ovary** | **0.0165** | **0.1320** |
| **Testes** | **0.0629** | **0.5032** |
| LLI Wall | 0.0845 | 0.6760 |
| Small Intestine | 0.0166 | 0.1328 |
| ULI Wall | 0.0871 | 0.6968 |
| Pancreas | 0.0330 | 0.2640 |
| Adrenal | 0.0221 | 0.1768 |
| Lung | 0.1320 | 1.0560 |
| Spleen | 0.0838 | 0.6704 |
| Brain | 0.1060 | 0.8480 |
| Heart | 0.0296 | 0.2368 |
| Urinary Bladder | 0.1960 | 1.5680 |
| Kidney | 0.1550 | 1.2400 |
| Liver | 0.1220 | 0.9760 |
| Gallbladder | 0.0378 | 0.3024 |
|  | **rem/mCi** | **rem/dose** |
| **Effective Dose** | **0.0631** | **0.5048** |

***** We thank Prof. Nabeel Nabulsi, Prof. Richard Carson and their colleagues at the Yale PET Center for generously allowing inclusion of their [^18^F]ASEM dosimetry in this article.

**3.2 [^11^C]Butanol Dosimetry**

| **Dose:** | **555 GBq / 15.0 mCi** | |
| --- | --- | --- |
| **Organ** | **rad/mCi** | **rad dose** |
| Adrenal | 0.0112 | **0.168** |
| Bladder Wall | 0.0114 | **0.171** |
| Stomach wall | 0.0115 | **0.173** |
| ULI Wall | 0.0266 | **0.399** |
| LLI Wall | 0.0134 | **0.201** |
| Kidney | 0.0156 | **0.234** |
| Liver | 0.0171 | **0.257** |
| Lung | 0.0095 | **0.142** |
| **Red Marrow** | **0.0092** | **0.138** |
| Muscle | 0.0097 | **0.145** |
| **Ovary** | **0.0146** | **0.219** |
| Pancreas | 0.0120 | **0.180** |
| Skin | 0.0078 | **0.117** |
| Spleen | 0.0187 | **0.281** |
| **Testes** | **0.0092** | **0.138** |
| Thyroid | 0.0094 | **0.142** |
| Uterus | 0.0142 | **0.213** |
| Total Body | 0.0106 | **0.159** |
| **Lens of Eye**** | **0.0078** | **0.117** |
| Brain | 0.0062 | **0.092** |
|  | **rem/mCi** | **rem/dose** |
| **Effective Dose (rem/mCi)** | **0.0160** | **0.240** |

**3.3 [^11^C]HED Dosimetry**

|  |  |  |  |  |
| --- | --- | --- | --- | --- |
| **Dose** | **740 MBq / 20 mCi** | | |  |
| **Organ** | **rad/mCi** | **rad/dose** |  |  |
| Whole Body | 0.0094 | **0.19** | |  |
| Lens of Eye | 0.0094 | **0.19** | |  |
| Red Marrow | 0.0081 | **0.16** | |  |
| Ovary | 0.0443 | **0.89** | |  |
| Testes | 0.0215 | **0.43** | |  |
| LLI Wall | 0.0076 | **0.15** | |  |
| Small Intestine | 0.0089 | **0.18** | |  |
| ULI Wall | 0.0094 | **0.19** | |  |
| Pancreas | 0.0454 | **0.91** | |  |
| Adrenal | 0.0311 | **0.62** | |  |
| Lung | 0.0104 | **0.21** | |  |
| Spleen | 0.0227 | **0.45** | |  |
| Brain | 0.0058 | **0.12** | |  |
| Heart | 0.0571 | **1.14** | |  |
| Urinary Bladder | 0.0074 | **0.15** | |  |
| Kidney | 0.0568 | **1.14** | |  |
| Liver | 0.0680 | **1.36** | |  |
| Gallbladder | 0.0162 | **0.32** | |  |
|  | **rem/mCi** | **rem/dose** |  |  |
| **Effective Dose Equivalent** | 0.0302 | **0.604** | |  |
| **Effective Dose** | 0.0199 | **0.398** | |  |

**3.4 [^11^C]LY2795050 Dosimetry***

| **DOSE** | **555 MBq / 15 mCi** | | |
| --- | --- | --- | --- |
| **Organ** | **rem/mCi** | **rem/dose** | **mSv/dose** |
| Adrenals | 1.88E-02 | **0.28** | **2.8** |
| Brain | 1.59E-02 | **0.24** | **2.4** |
| Breasts | 7.73E-02 | **1.16** | **11.6** |
| Gallbladder Wall | 2.19E-01 | **3.29** | **32.9** |
| Left colon (LLI Wall) | 7.53E-03 | **0.11** | **1.1** |
| Small Intestine | 1.05E-02 | **0.16** | **1.6** |
| Stomach Wall | 9.20E-03 | **0.14** | **1.4** |
| Right colon (ULI Wall) | 1.71E-02 | **0.26** | **2.6** |
| Heart Wall | 2.24E-02 | **0.34** | **3.4** |
| Kidneys | 3.63E-02 | **0.54** | **5.4** |
| Liver | 1.72E-01 | **2.58** | **25.8** |
| Lungs | 3.67E-02 | **0.55** | **5.5** |
| Muscle | 8.22E-03 | **0.12** | **1.2** |
| Ovaries | 1.08E-02 | **0.16** | **1.6** |
| Pancreas | 1.82E-02 | **0.27** | **2.7** |
| Red Marrow | 1.14E-02 | **0.17** | **1.7** |
| Osteogenic Cells | 1.41E-02 | **0.21** | **2.1** |
| Spleen | 2.29E-02 | **0.34** | **3.4** |
| Testes | 8.80E-03 | **0.13** | **1.3** |
| Thymus | 1.36E-02 | **0.20** | **2.0** |
| Thyroid | 1.47E-02 | **0.22** | **2.2** |
| Urinary Bladder Wall | 8.35E-03 | **0.13** | **1.3** |
| Uterus | 1.34E-02 | **0.20** | **2.0** |
|  |  |  |  |
| **Effective Dose:** | 2.17E-02 | **0.33** | **3.3** |

***** We thank Prof. Nabeel Nabulsi, Prof. Richard Carson and their colleagues at the Yale PET Center for generously allowing inclusion of their [^11^C]LY2795050 dosimetry in this article.

**3.5 [^11^C]MPH**

|  |  |  |  |  |
| --- | --- | --- | --- | --- |
| **Dose** | **666 MBq / 18 mCi** | | |  |
| **Organ** | **rad/mCi** | **rad/dose** |  |  |
| Whole Body | 0.010 | **0.180** | |  |
| Lens of Eye | 0.018 | **0.414** | |  |
| Red Marrow | 0.009 | **0.162** | |  |
| Ovary | 0.074 | **1.332** | |  |
| Testes | 0.118 | **2.124** | |  |
| LLI Wall | 0.017 | **0.306** | |  |
| Small Intestine | 0.082 | **1.476** | |  |
| ULI Wall | 0.016 | **0.288** | |  |
| Adrenal | 0.018 | **0.324** | |  |
| Lung | 0.014 | **0.252** | |  |
| Spleen | 0.029 | **0.522** | |  |
| Brain | 0.015 | **0.270** | |  |
| Heart | 0.012 | **0.216** | |  |
| Urinary Bladder Wall | 0.128 | **2.304** | |  |
| Kidney | 0.056 | **1.008** | |  |
| Liver | 0.033 | **0.594** | |  |
|  | **rem/mCi** | **rem/dose** |  |  |
| **Effective Dose Equivalent** | 0.052 | **0936** | |  |

**3.6 [^18^F]MPPF Dosimetry**

| **Dose:** | **259 MBq / 7.0 mCi** | |  |
| --- | --- | --- | --- |
| **Organ** | **Rad/mCi** | **Rad/Dose** |  |
| Whole Body | 4.8E-02 | **0.33** |  |
| Lens of Eye | 1.2E-02 | **0.08** |  |
| Red Marrow | 4.4E-02 | **0.31** |  |
| Ovary | 1.7E-01 | **1.17** |  |
| Testes | 2.0E-02 | **0.16** |  |
| LLI Wall | 2.4E-01 | **1.71** |  |
| Small Intestine | 6.6E-01 | **4.61** |  |
| ULI Wall | 7.1E-01 | **4.97** |  |
| Pancreas | 5.2E-02 | **0.36** |  |
| Adrenal | 5.9E-02 | **0.41** |  |
| Lung | 2.9E-02 | **0.20** |  |
| Spleen | 3.8E-02 | **0.27** |  |
| Brain | 1.2E-02 | **0.08** |  |
| Heart | 3.1E-02 | **0.22** |  |
| Urinary Bladder | 3.6E-01 | **2.53** |  |
| Kidney | 6.4E-02 | **0.45** |  |
| Liver | 8.1E-02 | **0.56** |  |
| Gallbladder | 9.7E-02 | **0.68** |  |
|  | **rem/mCi** | **Rem/dose** |  |
| **Effective Dose Equivalent** | 1.8E-01 | **1.27** |  |
| **Effective Dose** | 1.2E-01 | **0.87** |  |

**3.7 [^11^C]PMP Dosimetry**

| **Dose:** | **666 MBq / 18.0 mCi** | |  |
| --- | --- | --- | --- |
| **Organ** | **Rad/mCi** | **Rad/Dose** |  |
| Whole Body | 5.9E-03 | **0.11** |  |
| Lens of Eye | 5.9E-03 | **0.11** |  |
| Red Marrow | 4.6E-03 | **0.08** |  |
| Ovary | 5.3E-02 | **0.95** |  |
| Testes | 5.4E-02 | **0.97** |  |
| LLI Wall | 4.3E-03 | **0.08** |  |
| Small Intestine | 5.2E-03 | **0.09** |  |
| ULI Wall | 5.6E-03 | **0.10** |  |
| Adrenal | 1.9E-02 | **0.34** |  |
| Lung | 8.4E-03 | **0.15** |  |
| Spleen | 1.4E-02 | **0.25** |  |
| Brain | 5.0E-03 | **0.09** |  |
| Heart | 8.5E-03 | **0.15** |  |
| Urinary Bladder | 3.7E-01 | **0.66** |  |
| Kidney | 1.3E-01 | **2.25** |  |
| Liver | 5.5E-02 | **1.56** |  |
|  | **rem/mCi** | **rem/dose** |  |
| **Effective Dose Equivalent** | 3.1E-02 | **0.56** |  |
|  |  |  |  |

**3.8 [^11^C]RO-54864 Biodistribution***

| **Dose:** | **2 min (n = 2)** | | **5 min (n = 2)** | | **20 min (n=4)** | | **40 min (n=4)** | | **110 min (n=2)** | |  |
| --- | --- | --- | --- | --- | --- | --- | --- | --- | --- | --- | --- |
| **Organ** | **%id/g** | **%id/organ** | **%id/g** | **%id/organ** | **%id/g** | **%id/organ** | **%id/g** | **%id/organ** | **%id/g** | **%id/organ** |  |
| Bone marrow | 0.014 ± 0.001 | 0.003 ± 0.002 | 0.038 ± 0.002 | 0.006 ± 0.001 | 0.51 ± 0.15 |  | 0.53 ± 0.13 | 2.25 ± 0.11 | 0.05 ± 0.01 |  |  |
| Kidney | 0.10 ± 0.02 | 0.74 ± 0.16 | 0.08 ± 0.02 | 0.57 ± 0.11 | 0.37 ± 0.02 | 2.64 ± 0.15 | 0.29 ± 0.02 | 7.04 ± 1.23 | 0.02 ± 0.02 | 0.20 ± 0.005 |  |
| Liver |  |  |  |  | 0.22 ± 0.02 | 7.38 ± 0.26 | 0.20 ± 0.03 | 0.83 ± 0.15 |  |  |  |
| Spleen |  |  |  |  | 0.46 ± 0.02 | 0.85 ± 0.08 | 0.39 ± 0.06 | 0.83 ± 0.15 |  |  |  |
| Heart |  |  |  |  | 0.38 ± 0.04 | 1.17 ± 0.08 | 0.33 ± 0.04 | 0.87 ± 0.02 |  |  |  |
| Lung |  |  |  |  | 0.35 ± 0.09 | 1.33 ± 0.18 | 0.23 ± 0.03 | 1.30 ± 0.17 |  |  |  |
| Muscle leg |  |  |  |  | 0.05 ± 0.01 |  | 0.05 ± 0.005 |  |  |  |  |
| Pancreas |  |  |  |  | 0.25 ± 0.04 | 0.47 ± 0.22 | 0.23 ± 0.05 | 0.53 ± 0.43 |  |  |  |
| Brain |  |  |  |  | 0.14 ± 0.01 | 0.75 ± 0.06 | 0.13 ± 0.02 | 0.70 ± 0.12 |  |  |  |
| Testes |  |  |  |  | 0.16 ± 0.02 | 1.35 ± 0.20 | 0.17 ± 0.01 | 1.48 ± 0.06 |  |  |  |
| Eyeball |  |  |  |  | 0.04 ± 0.01 | 0.03 ± 0.005 | 0.04 ± 0.01 | 0.035 ± 0.004 |  |  |  |
| Adipose |  |  |  |  | 0.14 ± 0.04 |  | 0.20 ± 0.04 |  |  |  |  |
| Urine |  |  |  |  | 0.009 ± 0.003 | 0.005 ± 0.005 | 0.007 ± 0.005 | 0.004 ± 0.00 |  |  |  |
| Blood |  |  |  |  | 0.03 ± 0.005 |  | 0.02 ± 0.001 |  |  |  |  |

***** Historical dosimetry data is no longer extant. Biodistribution data are provided to enable estimation of dosimetry.

1. **Rationale for no mass dose limit in certain cases**

The following radiotracers do not have formal mass dose limits established because they are generally recognized as safe and effective or they are found in the human body at levels far greater than subjects receive from a radiotracer microdose administered during a PET scan (e.g. endogenous compounds, dietary components or essential nutrients).

**[^11^C]Acetate**

It is estimated that the amount of acetate in humans is about 50–60 μmol/L (3.0–3.6 mg/L) in plasma and 116 μmol/L (7 mg/L) in cerebrospinal fluid. The estimated turnover of acetate is about 45 g/d (Pravasi, 2014).

**[^13^N]Ammonia**

Ammonia is a waste product generated during digestion of protein. It is processed by the liver, where it is converted to urea and eliminated in the urine. Normal blood levels of ammonia are ≤35 µmol/L (Khan *et al*., 2016).

**[^11^C]Choline**

Choline is obtained from dietary sources and serves as a precursor for biosynthesis of acetylcholine and phospholipids amongst other things. The primary criterion used to estimate the Adequate Intake (AI) for choline is the prevention of liver damage (determined from serum alanine aminotransferase levels). For adults, the recommended amount of choline is 550 mg/day for men and 425 mg/day for women. The Tolerable Upper Intake Level is 3.5 g/day (Institute of Medicine, 1998).

**[^18^F]FDG**

FDG is the most widely utilized PET radiotracer, with millions of scans occurring annually around the world. FDG is generally recognized as safe and effective and is manufactured by no-carrier-added methods. As such, there are no mass dose limits for FDG and testing for molar activity is not required (Hung, 2002).

**[^11^C]Methionine**

The Food and Nutrition Board of the U.S. Institute of Medicine established Recommended Dietary Allowances (RDAs) for essential amino acids in 2002. The RDA for methionine (combined with cysteine) has been set at 14 mg/kg per day for adults (Nimni *et al.*, 2007). Note, the European Pharmacopeia sets a limit of maximum 2.0 mg of methionine per maximum recommended dose in milliliters (L-Methionine ([^11^C]methyl) injection, 2020).

**[^11^C]Palmitate**

Palmitic acid is the most common saturated fatty acid found in the human body and can be obtained from dietary sources or biosynthesized by the body from other fatty acids, carbohydrates and amino acids. Palmitic acid represents approximately 20–30% of total fatty acids in membrane phospholipids and adipose triacylglycerols and an average 70 kg male consists of ~3.5 kg of palmitic acid (Carta *et al*., 2017).

**[^11^C]Sarcosine**

Sarcosine (N-methyl glycine) is an amino acid derivative that naturally occurs in muscles and other tissues. The concentration of sarcosine in blood serum of normal human subjects has been reported as 1.4 ± 0.6 µM (Allen *et al*.,1997).

**[^18^F]Sodium fluoride**

The beneficial effects of fluoride on human health are well known, including reduction in the prevalence of dental decay and formation of stronger teeth and bones. The recommended level of fluoride in drinking water varies from 0.7 mg/L – 1.5 mg/L, while the U.S. National Academy of Sciences Institute of Medicine has recommended an AI for fluoride of 0.05 mg/kg/day (Institute of Medicine, 1997; Ullah *et al*., 2017). Note, the European Pharmacopeia sets a limit of maximum 4.52 mg of fluoride per maximum recommended dose in milliliters (Sodium Fluoride (^18^F) Injection, 2020).

**[^15^O]Water**

Up to 60% of the human adult body is water and so no justification is required.

1. **References**

Allen RH, Stabler SP, Lindenbaum J. Serum betaine, N,N-dimethylglycine and N-methylglycine levels in patients with cobalamin and folate deficiency and related inborn errors of metabolism. Metabolism. 1993;42:1448–60.

Carta G, Murru E, Banni S, Manca C. Palmitic Acid: Physiological Role, Metabolism and Nutritional Implications. Front Physiol. 2017;8:902.

Connelly, J. (2018) ICH Q3C Impurities: Guideline for Residual Solvents. In ICH Quality Guidelines by A. Teasdale, D. Elder and R. W. Nims (Eds). John Wiley & Sons, Inc. pp 199-232.

Gao Y, Kellar KJ, Yasuda RP, Tran T, Xiao Y, Dannals RF, Horti AG. Derivatives of dibenzothiophene for PET imaging of a7-nicotinic acetylcholine receptors. J Med Chem. 2013;56:7574–7589.

Hung JC. Comparison of Various Requirements of the Quality Assurance Procedures for ^18^F-FDG Injection. J Nucl Med. 2002;43:1495–1506.

Institute of Medicine (US) Standing Committee on the Scientific Evaluation of Dietary Reference Intakes. Washington (DC): National Academies Press (US); 1997.

Institute of Medicine (US) Standing Committee on the Scientific Evaluation of Dietary Reference Intakes and its Panel on Folate, Other B Vitamins, and Choline. Washington (DC): National Academies Press (US); 1998.

Khan A, Ayub M, Khan WM. Hyperammonemia Is Associated with Increasing Severity of Both Liver Cirrhosis and Hepatic Encephalopathy. Int J Hepatol. 2016; 2016: 6741754.

L-Methionine ([^11^C]methyl) injection. European Pharmacopoeia 2020;10.0:1220.

Mossine A, Brooks AF, Jackson IM, Quesada CA, Sherman P, Cole EL, Donnelly DJ, Scott PJH, Shao X. Synthesis of Diverse ^11^C-Labelled PET Radiotracers via Direct Incorporation of [^11^C]CO_2_. Bioconj Chem*.* 2016;27:1382-1389.

Mossine AV, Tanzey SS, Brooks AF, Makaravage KJ, Ichiishi N, Miller JM, Henderson BD, Skaddan M, Sanford MS, Scott PJH.: One-pot Synthesis of High Molar Activity 6-[^18^F]Fluoro-L-DOPA by Cu-Mediated Fluorination of a BPin Precursor Org. Biomol. Chem. 2019;17:8701-8705.

Mossine AV, Tanzey SS, Brooks AF, Makaravage KJ, Ichiishi N, Miller JM, Henderson BD, Skaddan MB, Sanford MS, Scott PJH.: Synthesis of High Molar Activity 6-[^18^F]Fluoro-L-DOPA Suitable for Human Use by Cu-Mediated Fluorination of a BPin Precursor Nat. Prot. 2020;15:1742-1759.

Nimni ME, Han B, Cordoba F. Are we getting enough sulfur in our diet? Nutr Metab (Lond). 2007; 4: 24.

Pravasi DS. Acetic Acid. In Encyclopedia of Toxicology (Third Edition). 2014; pp 33-35.

Rodnick, M. E.; Sollert, C.; Stark, D.; Clark, M.; Katsifis, A.; Hockley, B. G.; Parr, D. C.; Frigell, J.; Henderson, B. D.; Abghari-Gerst, M.; Piert, M. R.; Fulham, M. J.; Eberl, S.; Ggnon, K.; Scott, P. J. H. Cyclotron-based Production of ^68^Ga, [^68^Ga]GaCl_3_, and [^68^Ga]Ga-PSMA-11 from a Liquid Target EJNMMI Radiopharm Chem, **2020**, *submitted (Preprint available here:* <https://www.researchsquare.com/article/rs-38981/v1>, DOI: 10.21203/rs.3.rs-38981/v1*)*.

Scott PJH, Kilbourn MR. Determination of residual Kryptofix 2.2.2 levels in [^18^F]-labeled radiopharmaceuticals for human use. Appl. Radiat. Isot. 2007;65:1359-1362.

Sodium Fluoride (^18^F) Injection. European Pharmacopoeia 2020;10.0:1227.

Ullah R, Zafar MS, Shahani N. Potential fluoride toxicity from oral medicaments: A review. Iran J Basic Med Sci. 2017;20:841–848.
